# Non-coding RNAs as regulators of the Hippo pathway in cardiac development and cardiovascular disease

**DOI:** 10.3389/fphar.2024.1348280

**Published:** 2024-04-18

**Authors:** Mengyang Song, He Wang, Caixia Liu, Sijie Jin, Bin Liu, Wei Sun

**Affiliations:** ^1^ Department of Cardiology, The Second Hospital of Jilin University, Changchun, China; ^2^ Department of Neurology, The Liaoning Province People’s Hospital, Shenyang, China

**Keywords:** microRNA, lncRNA, circRNA, Hippo pathway, cardiovascular development, cardiovascular disease

## Abstract

Cardiovascular diseases pose a serious threat to human health. The onset of cardiovascular diseases involves the comprehensive effects of multiple genes and environmental factors, and multiple signaling pathways are involved in regulating the occurrence and development of cardiovascular diseases. The Hippo pathway is a highly conserved signaling pathway involved in the regulation of cell proliferation, apoptosis, and differentiation. Recently, it has been widely studied in the fields of cardiovascular disease, cancer, and cell regeneration. Non-coding RNA (ncRNAs), which are important small molecules for the regulation of gene expression in cells, can directly target genes and have diverse regulatory functions. Recent studies have found that ncRNAs interact with Hippo pathway components to regulate myocardial fibrosis, cardiomyocyte proliferation, apoptosis, and hypertrophy and play an important role in cardiovascular disease. In this review, we describe the mode of action of ncRNAs in regulating the Hippo pathway, provide new ideas for further research, and identify molecules involved in the mechanism of action of ncRNAs and the Hippo pathway as potential therapeutic targets, with the aim of finding new modes of action for the treatment and prevention of cardiovascular diseases.

## 1 Introduction

Cardiovascular disease (CVD) is one of the main diseases that affects human health. According to the latest data from the American Heart Association, cardiovascular disease is currently the main cause of death globally, which presents a great economic burden to society worldwide (Tsao et al., 2023) therefore, it is important to delve into the pathophysiological molecular mechanisms involved in cardiovascular diseases. The Hippo pathway is crucial in heart development and homeostasis. Components of the Hippo pathway were first identified as tumor suppressors in *Drosophila melanogaster* (Justice et al., 1995). Recent research has found that the Hippo pathway and its downstream transcriptional co-activators YAP/TAZ play crucial roles in environmental homeostasis, organ size, tissue regeneration, and stem cell function (Johnson and Halder, 2014; Panciera et al., 2017; Dey et al., 2020). Dysfunction of the Hippo pathway can cause changes in the cardiac structure and function, leading to diseases such as heart failure, cardiomyopathy, coronary heart disease, arrhythmia, and cardio-oncology disease (Xin et al., 2013; Zhou et al., 2015; Wang et al., 2018). Therefore, the Hippo pathway is a promising therapeutic target for cardiovascular diseases. Many small-molecule compounds targeting the Hippo pathway have been developed, among which non-coding RNA (ncRNA) is expected to become a new treatment for cardiovascular diseases.

NcRNA was initially considered to be a form of “genetic noise” without practical functions, transcribed from the evolutionarily unrelated DNA and non-coding sequences in the genome (Struhl, 2007). However, recent studies have found that ncRNA can participate in the regulation of a variety of physiological and pathological processes by regulating the transcription and translation of genes, and plays important roles in cancer, cardiovascular diseases, neurological diseases, and other diseases (Beermann et al., 2016; Li et al., 2021; Chen et al., 2022). Among ncRNAs, miRNA, circRNA, and lncRNA are the most widely studied and mature ncRNAs and have been found to play important roles in cardiovascular function and dysfunction. They not only participate in heart development and disease occurrence and development, but also can be used as potential biomarkers for disease prediction and monitoring (Beermann et al., 2016; Rai et al., 2021; Zhang et al., 2021). In addition, ncRNA can be delivered to cells and extracellular environments as drugs to play a role in treating and preventing diseases (Shah and Giacca, 2022). Therefore, studying the role of these small molecules in the body is crucial and can provide strategies for advancing drug development and clinical application. In recent years, an increasing number of studies have revealed the molecular mechanisms by which ncRNAs participate in the regulation of the Hippo pathway in cardiovascular diseases, providing new ideas for the prevention and treatment of cardiovascular diseases.

In this review, we discuss the important role of the Hippo pathway in heart development and cardiovascular disease and further summarize the known ncRNAs, especially miRNAs, circRNAs, and lncRNAs that play a role in cardiovascular disease by regulating the Hippo pathway to provide feasible ideas and strategies for the treatment of cardiovascular disease and cardiac regeneration.

## 2 Hippo signaling pathway

The Hippo pathway primarily plays a role in growth control, tumor inhibition, and the promotion of cell apoptosis, which are crucial for controlling organ size and body homeostasis. The core components of Hippo signaling in mammals include Mammalian sterile 20-like 1/2 (MST1/2, also called STK4/3), Salvador (SAV1), Large tumor suppressor homolog 1/2 (LATS1/2), MOB kinase activator 1A/B (MOB1a/b), Yes-associated protein (YAP)/transcriptional co-activator with PDZ binding motif (TAZ, also called WWTR1), Vestigial-like family member 4 (VGLL4), and transcription factors TEAD family of transcription factors (TEAD1-4) (Yu et al., 2015).

When mammalian cells receive signals from both intracellular and extracellular stimuli, such as cell contact inhibition, cell adhesion and polarity, cytoskeletal remodeling, osmotic pressure, cell energy status, soluble factors, mechanical forces, stress signals, bacterial infection, and G protein-coupled receptors (GPCR) ligand signals, positive or negative upstream regulators of the Hippo pathway recognize these signals at the plasma membrane and transmit them to the core kinase cascade of the Hippo pathway. However, the mechanisms by which these upstream regulators respond to various input signals remain unclear (Yu et al., 2015; Moya and Halder, 2019; Zheng and Pan, 2019; Wang et al., 2022).

**FIGURE 1:**
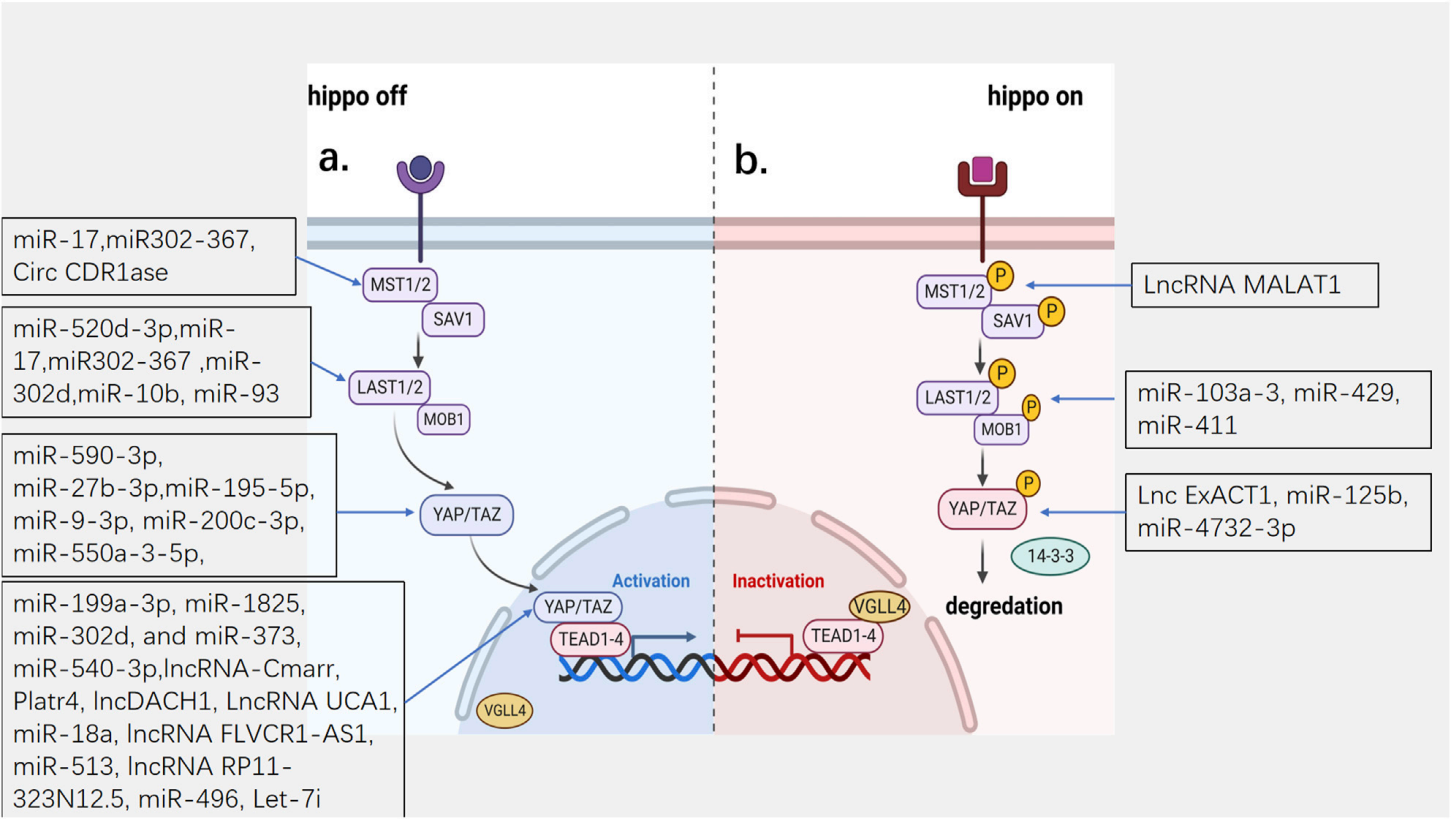
The mechanism of Hippo pathway and the hippo targets of ncRNAs. **(A)** the Hippo pathway is inactivated. YAP and TAZ cannot enter the nucleus and are subsequently degraded by the proteasome. **(B)** the Hippo pathway is activated. YAP/TAZ enters and accumulates in the nucleus and promote the transcription of genes. MST1/2: Mammalian sterile 20-like 1/2; SAV1: Salvador; LATS1/2:Large tumor suppressor homolog 1/2; MOB1a/b:MOB kinase activator 1A/B; YAP:Yes-associated protein; TAZ: transcriptional co-activator with PDZ binding motif; VGLL4:Vestigial-like family member 4; TEAD1-4: transcription factors TEAD family.

The Hippo pathway is activated or closed by various upstream regulatory factors and environmental signals. When the core kinase cascade of the Hippo pathway is activated, MST1/2 and its cofactor SAV become phosphorylated and further phosphorylate the downstream protein LATS1/2 and its scaffolding protein, Mob1. The hippo pathway becomes switched to the “on” state, and the phosphorylated LATS1/2 further phosphorylates YAP and TAZ, which consequently cannot enter the nucleus and bind to 14-3-3 protein in the cytoplasm and are subsequently degraded by the proteasome. VGLL4 competitively binds to TEAD via YAP and inhibits gene transcription. The kinase cascade becomes inactive when the upstream negative regulator acts on the kinase cascade, the Hippo pathway is inactivated, and YAP/TAZ enters and accumulates in the nucleus and cannot directly bind to DNA. Therefore, binds to the transcription factor TEAD instead to promote the transcription of genes related to cell proliferation, cell cycle, and fibrosis, which contributes to the occurrence of cancer and tissue repair and regeneration (Johnson and Halder, 2014; Yu et al., 2015; Misra and Irvine, 2018) (Figure 1).

Based on the above basic modes of action of the Hippo pathway, several aspects require research attention: 1. YAP/TAZ activity can be regulated by upstream inputs independent of the kinase cascade, which includes other kinases such as 5′AMP-activated protein kinase (AMPK), Abelson tyrosine-protein kinase (Abl), and the proto-oncogene tyrosine-protein kinase Src, as well as the actin cytoskeleton (Piccolo et al., 2014). 2. In addition to TEAD, nuclear YAP/TAZ has been shown to interact with other transcription factors, including Smad, RUNT-related transcription factor 1/2 (RUNX1/2), p63/p73, T-box transcription factor 5 (TBX5), and ErbB4 (Johnson and Halder, 2014; Varelas, 2014). 3. The expression of VGLL4 is repressed by miR-130a, which is directly induced by YAP, leading to the amplification of YAP activity (Shen et al., 2015). 4. YAP/TAZ exists in both the nucleus and cytoplasm, and its distribution is not static but constantly shuttles between the nucleus and cytoplasm. The cellular location of YAP/TAZ and its dominant distribution in the nucleoplasm depend on its relative rate of nuclear input and output, which depends on its degree of phosphorylation, cell type, context environment, and other factors affecting nuclear output and nuclear input (Manning et al., 2020). As the transcriptional function of YAP determines cell fate and the function of the Hippo pathway, it is important to pay attention to its distribution and related regulatory factors.

## 3 Non-coding RNA

ncRNA previously was considered as evolutionary “junk” (Beermann et al., 2016). In 2012, the Encyclopedia of DNA Elements (ENCODE) project unveiled the human genome. It revealed that less than 2% of the human genome encodes proteins and that most of the remaining sequences appear to participate in biochemical activities that may be functionally important (Encode, 2012). Moreover, the emergence of gene sequencing tools, such as RT-PCR (reverse transcription-polymerase chain reaction), next-generation sequencing, and microarray analysis, has made it possible to rapidly sequence nucleic acids at a large scale, and thus they are considered powerful tools for studying the specific characteristics of ncRNA and related diseases (Loganathan and Doss, 2023).

**FIGURE 2:**
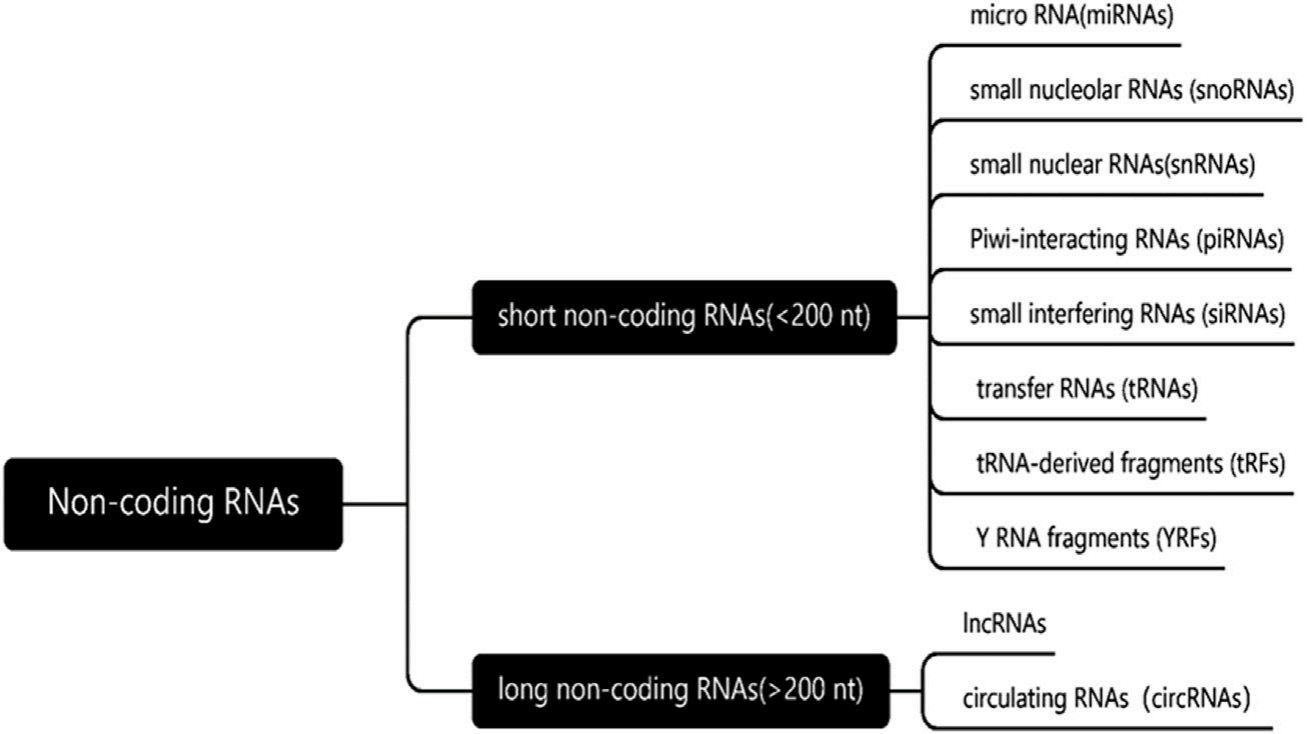
Classification of non-coding RNAs. According to the length and mechanism of ncRNA, the non-coding RNAs can be divided into two categories, length of less than 200 nucleotides and more than 200 nucleotides in length.

As we all know, according to the length and mechanism of ncRNA, it can be divided into two categories (Figure 2). The first is a short non-coding RNA with a length of less than 200 bp, which mainly includes micro-RNA (miRNAs), small nucleolus RNAs (SnoRNAs), micronucleus RNAs, Piwi-interacting RNAs (PiRNAs), small interference RNAs (SiRNAs), transfer RNAs (tRNAs), tRNA-derived fragment (TRFs) and YRNA fragment (YRNAs). This class is represented by miRNA, which regulates gene expression by directly acting on mRNA. The second type is long-chain noncoding (lncRNAs) and circular RNA (circRNAs) with a length of more than 200 bp, which regulate gene transcription by acting on miRNA indirectly or directly on mRNA. In this article, we will mainly discuss miRNAs, lncRNAs and CircRNAs, which are the most widely studied at present (Ali et al., 2021).

miRNAs are short-chain (approximately 21–25 nucleotides) single-stranded ncRNA. In most cases, they target the 3′untranslated region (3′UTR) of mRNA through RNA silencing complex (RISC), leading to mRNA degradation and translation inhibition (O'Brien et al., 2018; Donde et al., 2022). In rare cases, miRNAs can regulate gene expression by inducing translation activation and transcriptional regulation (O'Brien et al., 2018). In addition, miRNAs can be secreted into the extracellular and circulatory systems to maintain regulatory activity, thus serving as intercellular communication signals and potential disease biomarkers (O'Brien et al., 2018).

CircRNA is a special ring long chain ncRNA, which is formed by covalent closure of the 5′and 3′ends, lacks a cap and tail, and is largely conserved between species, especially between mice and humans (Brozzi and Regazzi, 2021; Sygitowicz and Sitkiewicz, 2022). CircRNA is considered primarily a miRNA “sponge,” serving as a competitive inhibitor that contains multiple miRNA binding sites, preventing miRNA from binding to mRNA and inhibiting the degradation of mRNA (Sygitowicz and Sitkiewicz, 2022). In addition, CircRNA can also act on RNA binding proteins (RBPs) as a “sponge” to regulate gene transcription and translation (Sygitowicz and Sitkiewicz, 2022).

LncRNAs are a kind of ncRNA that is spliced, capped, polyadenylated and transcribed by RNA polymerase II, with a length of more than 200 nucleotides. It is highly conserved and exhibits tissue specificity. It has a highly conserved function, but its sequence is usually not conserved between species. Furthermore, the ambiguity of its characteristics and high instability increase the complexity of its function and classification, which has greatly challenged research into this type of ncRNA, making it the most complex type of ncRNA (Andergassen and Rinn, 2022; Chen et al., 2022; Nojima and Proudfoot, 2022). The subcellular localization of lncRNAs is closely related to their functions. Most lncRNAs are localized in the nucleus. They can change the chromatin environment by directly binding to DNA or interacting with proteins, such as chromatin-modifying proteins, mediating gene silencing or activation, and regulating gene expression and DNA repair. Additionally, lncRNAs located in the nucleus participate in transcriptional regulation. LncRNAs are crucial for the assembly and function of nuclear aggregates and play a role in nuclear stability (Statello et al., 2021). LncRNAs can also be secreted into the cytoplasm, and some directly bind to proteins to form lncRNA protein complexes (lncRNPs), leading to changes in mRNA splicing and conversion. In addition, lncRNAs can serve as sponges for miRNAs, preventing their binding to mRNA. lncRNA found in the cytoplasm may also originate from organelles, where lncRNA in exosomes enter receptor cells through secretion into the extracellular and circulatory systems and participate in epigenetic regulation, cell-type reprogramming, and genomic instability. Furthermore, lncRNA in mitochondria is closely associated with metabolism, apoptosis, and the interactions between mitochondria and nuclei (Statello et al., 2021).

## 4 ncRNA/Hippo pathway in cardiac development

The Hippo pathway is crucial for heart development and size control. Heart development in embryos begins in the mesoderm, and heart size depends on the increase in the number of cells during embryonic development. Studies have shown that the Hippo pathway plays an indispensable role in embryonic heart development (Table 1).

**TABLE 1:** Hippo signaling kinases and mediators involved in cardiac development.

Gene	Animal model/cell species	Mechanism	Results	Ref.
YAP	hESCs	YAP ↓→WNT3↑→ SMAD2,3↑→ Sox17, Foxa2, and Cer1↑	Promote the differentiation of cardiomyocyte lineage	Hsu et al. (2018)
YAP	Rat/cardiomyocytes	YAP↑ → CcnA2, CcnB1, Cdc2, Ect2↑ and Cabc1, Dapk↓	Cardiomyocyte proliferation is significantly increased, leading to drastic overgrowth of the myocardium and expansion of myocardial trabeculae	von Gise et al. (2012), Xin et al. (2011)
YAP	Mouse/cardiomyocytes	YAP↑→IGF↑→ GSK-3β↓→ β-catenin↑→Kcne3, Ndrl, and Ier3 ↑	Increase the number of cardiomyocytes and heart size and promote cardiomyocyte proliferation	Xin et al. (2011), Lin et al. (2015)
		YAP↑→Pik3cb↑→PI3K-AKT signaling↑		
YAP	hESC-CMs	Nuclear YAP↑→EGFR↑	Promote cardiomyocyte proliferation	Park et al. (2018)
YAP	Mouse/cardiomyocytes	PGC1/PPAR↑→YAP↑	Promote cardiomyocyte hypertrophy and maturation	Murphy et al. (2021)
YAP	Zebrafish/Endoderm Cells	S1P-S1pr2→p-lats1/2↓→nuclear YAP-TEAD↑ →Ctgfa↑	Ensure the formation of the endoderm and cardiac tubes	Fukui et al. (2014)
YAP	Mouse/cardiomyocytes	p-YAP-DAG1↑→nuclear YAP↓	Inhibit cardiomyocyte proliferation and regulate cardiac maturation	Morikawa et al. (2017)
YAP	Mouse neonatal heart/cardiomyocytes	α-catenin, Fat4→YAP cytoplasmic localization↑	Inhibit postnatal cardiomyocyte proliferation	Ragni et al. (2017), Vite et al. (2018)
YAP	Mouse heart/cardiomyocytes	cell cycle: Ccne2 and Cdk6	Promote cardiomyocytes to sense external mechanical forces; transmit external signals into cells; and promote cell division, proliferation, and migration	Morikawa et al. (2015)
		Cell division: Aurkb, Birc5, Spin1, and Aspm		
		Cytoskeleton formation and migration: Enah, Fgd4, and Mtss1		
		The cytoskeleton connects the extracellular matrix: Ctnna3, Sgcd, and Tln2		
TAZ	Zebrafish/cardiomyocytes	TAZ↓→tp1-Notch↓	Inhibit the proliferation and skeleton maturation of cardiomyocytes and inhibit the formation of trabeculae	Lai et al. (2018)
		TAZ↓→myh10↓, mybphb↓		
YAP + TAZ	Mouse/epicardial cells	YAP + TAZ↓→Tbx18, Wt1↓	Affect epicardial EMT and differentiation into coronary endothelial cells and abnormal coronary artery development	Singh et al. (2016)
YAP + TAZ	Mouse/endocardial cells	YAP + TAZ↑→VE-cadherin	Promote endocardial cell proliferation	Artap et al. (2018), Bornhorst et al. (2019)
YAP + TAZ	Mouse/endocardial cells	YAP + TAZ↓→Nrg1-ErbB↓	Cause damage to endocardial cell EMT, myocardial wall thinning, and early *postpartum* death	Artap et al. (2018), Bornhorst et al. (2019)
YAP/TAZ	zebrafish/cells within the anterior lateral plate mesoderm (ALPM)	Yap/TAZ↓→Bmp2b, hand2↓	Limit the number of CPCs in the SHF at the venous pole, limiting the size of the atria	Francou et al. (2017), Fukui et al. (2018)
LATS1/2	Mouse/embryonic cells	p-LATS1/2 ↑→p-Paptor S606 ↑→ mTORC1↓	Limit embryonic heart size	Gan et al. (2020a)
LATS1/2	Mouse/embryonic cardiomyocytes	LATS1/2↓→nuclear YAP↑→YAP-TEAD↑→Dpp4, Dhrs3↓	Cause defects in the differentiation of subepicardial C20 cells into fibroblasts and defects in coronary vascular development	Xiao et al. (2018)
LATS2	Mouse/cardiomyocytes	p-MST1 ↑→p-LATS2 ↑→Bcl-xL↓; LATS2↑→p-Akt↓, p-70S6K↓, p-eEF2↑	Promote cell apoptosis, inhibit protein synthesis, and limit the size of cardiomyocytes and ventricular cavities	Matsui et al. (2008)
TEAD	Rat/cardiomyocytes	TEAD↑→SRF, MEF2↑	Regulate cardiomyocyte gene expression	Chen et al. (1994), Gupta et al. (2001), Maeda et al. (2002)
Mst1/2	Mouse/embryonic stem cells (ES)	Mst1/2↓→p-YAP↓, nuclear YAP↑→Ctgf, Cyr61↑	Embryos proliferate faster; however, differentiation into cardiac progenitors and cardiomyocytes is blocked	Li et al. (2013)
		Mst1/2↓→Nanog↑, Wnt2/2b,Wnt5a↓		
VGLL4	Neonatal mouse heart/cardiomyocytes	VGLL4-TEAD↓→YAP-TEAD↑	Promotes the proliferation of cardiomyocytes in the neonatal heart	Lin et al. (2016)
VGLL4	Mature mouse heart/cardiomyocytes	VGLL4-TEAD↑→YAP-TEAD↓	Inhibits cardiomyocyte proliferation, promote necrosis, and related to cardiac maturation	Lin et al. (2016)

### 4.1 Hippo signaling kinases and mediators in cardiac development

#### 4.1.1 YAP

Deletion of the YAP gene resulted in the death of most rat embryos by lethal myocardial hypoplasia before E16.5, which was caused by a reduction in cardiac cell proliferation. Inhibition of YAP activity has been reported to regulate primitive streak (PS) differentiation in the anterior PS (APS), which is a precursor of the cardiac mesoderm (Hsu et al., 2018). Hsu et al. (Hsu et al., 2018) found that a loss of YAP expression caused hESCs to differentiate into APS in response to the regulation of ACTIVIN, accompanied by the upregulated expression of pan-PS and APS genes, such as *SOX17* (*SRY (sex determining region Y)-box 17*), and generated viable cardiomyocytes under the action of YAP inhibitors by constructing a YAP knockout model and using a mixture of YAPi molecules. Additional studies led us to hypothesize that the loss of YAP in hESCs activates endogenous WNT3 signaling and allows SMAD2, 3 to induce the expression of pan-PS and APS genes under the action of ACTIVIN-induced differentiation. Therefore, YAP plays an important role in regulating the differentiation of cardiomyocyte lineages. In addition, cardiac cell proliferation increases after overexpression of YAP *in vivo*, causing dramatic myocardial overgrowth and expansion of the trabecular myocardium, which requires the interaction of YAP-TEAD (von Gise et al., 2012). Analysis of related genes showed that YAP may promote the proliferation of cardiomyocytes by inducing the expression of *cyclin A2 (CcnA2), cyclin B1 (CcnB1), cyclin-dependent kinase 1 (Cdc2), and Ect2* and inhibiting the expression of the apoptosis-promoting genes *Cabc1 and death associated protein kinase (Dapk)* (von Gise et al., 2012; Xin et al., 2011). YAP may also promote cardiac proliferation by activating insulin-like growth factor (IGF), which results in the inactivation of glycogen synthase kinase 3b (GSK-3β), leading to increased expression of β-catenin, a positive regulator of cardiac growth. Additionally, the expression of *potassium voltage-gated channel subfamily E regulatory subunit 3*(*Kcne3*), *N-myc downstream regulated gene 1* (*Ndrg1*), and *immediate early response 3*(*Ier3*), which are regulated by β-catenin, is also significantly increased by YAP, further indicating that YAP promotes the expression of β-catenin. YAP can also increase cardiomyocyte proliferation by promoting the activation of the PI3K-AKt signaling pathway, which is mediated by Pik3cb in cardiomyocytes (Xin et al., 2011; Lin et al., 2015). Furthermore, in human embryonic stem cell-derived CMs (hESC-CMs) cultured at a low density, an increase in nuclear YAP expression mediated epidermal growth factor receptor (EGFR) signaling, thereby increasing cardiomyocyte proliferation (Park et al., 2018).

Murphy et al. (Murphy et al., 2021) used Ingenuity Pathway Analysis (IPA) to analyze factors affecting cardiomyocyte maturation, and the results showed that transcription cofactors and a group of nuclear receptors (PPAR, thyroid hormone receptor, and retinol receptor) may be upstream transcription regulators. Further research confirmed that YAP, a component of cardiac hypertrophy, is a key regulator of PGC1/PPAR signal transduction in cardiomyocyte maturation (Murphy et al., 2021). In addition, in zebrafish, sphingosine 1-phosphate- S1P receptors2 (S1P-S1pr2) signaling induces YAP nuclear translocation and increases YAP/TEAD-dependent ctgfa expression via Lats1/2 inactivation in the endoderm, ensuring proper formation of the endoderm, which is required for the migration of cardiac precursor cells (CPCs) to the midline to form the cardiac tube (Fukui et al., 2014). The dystrophin glycoprotein complex (DGC) is a multi-component transmembrane complex that connects the actin cytoskeleton and the extracellular matrix, which is essential for CM homeostasis. DAG1, a component of DGC, directly binds to YAP to inhibit its nuclear translocation and transcriptional activity, which in turn inhibits the proliferation of cardiac myocytes and may play a role in the maturation of cardiac myocytes after birth. Hippo-induced YAP phosphorylation further enhances the Yap-DAG1 interaction (Morikawa et al., 2017). Therefore, Hippo-Yap-DGC forms a negative regulatory loop that prevents the postnatal proliferative response of the heart and regulates postnatal heart development. Moreover, Vite et al. (Vite et al., 2018) and Ragni (Ragni et al., 2017) found that the components of cell junctions, α-catenin and atypical cadherin Fat4, mediate the cytoplasmic localization of YAP in a non-hippo pathway dependent manner during the formation of mice neonatal cardiac cell junctions, inhibiting cell proliferation. This finding provides a new understanding for the loss of the proliferative ability of mammalian neonatal cardiac myocytes.

In addition, YAP target genes are preferentially expressed in fetal hearts and promote the proliferation of cardiomyocytes. They primarily comprise three categories: cell cycle (*cyclin E2*(*Ccne2*) and *cyclin dependent kinase 6*(*Cdk6*)) and cell division (*aurora kinase B* (*Aurkb*), *baculoviral IAP repeat containing 5*(*Birc5*), *spindlin 1*(*Spin1*), and *assembly factor for spindle microtubules* (*Aspm*)) genes; genes involved in cytoskeleton formation and cell migration (*ENAH actin regulator* (*Enah*), FYVE, *RhoGEF and PH domain containing 4*(*Fgd4*), and *MTSS I-BAR domain containing 1*(*Mtss1*)); and genes connecting the actin cytoskeleton to the extracellular matrix (*catenin alpha 3*(*Ctnna3*), *sarcoglycan delta* (*Sgcd*), and *talin 2*(*Tln2*)) which sense external mechanical forces, transmit external signals to cells, and promote cell migration, division, and proliferation (Morikawa et al., 2015).

#### 4.1.2 YAP + TAZ

Although TAZ deletion in the embryo does not result in any cardiac defects, it plays an essential role in the formation of cardiac trabeculation and maturation of both the ventricular chamber and cardiac wall. TAZ regulates cardiomyocyte expression of the tp1 Notch reporter gene in a cell-autonomous manner, promoting the maturation of cardiac trabeculae. Simultaneously, developing hearts with TAZ deficiency exhibit differentially expressed genes, among which, genes regulating the actomyosin network, including *myosin heavy chain 10(myh10)* and *myosin binding protein Hb* (*mybphb*), affect the maturation of trabeculae (Xin et al., 2013; Lai et al., 2018; He et al., 2022). The knockout of YAP/TAZ in the epicardium of mouse embryos is embryonically lethal, resulting in defective coronary arteries (Singh et al., 2016; Xiao et al., 2018). During embryonic epicardium development, YAP/TAZ promotes the development of the coronary artery by partially regulating the expression of *T-box transcription factor 18(Tbx18)* and *Wt1*, affecting the proliferation and migration of epicardium cells, epicardial epithelial-to-mesenchymal transition (EMT), and other processes (Singh et al., 2016). Artap et al. (Artap et al., 2018) and Bornhorst et al. (Bornhorst et al., 2019) investigated the role of Hippo signaling in the endocardium of developing hearts. They found that YAP/TAZ in the endocardium could respond to connective tension transduced by the endothelial-specific adherens junction protein cadherin-5 (VE-cadherin), resulting from the enlargement of the atrial volume, to promote the proliferation of endocardial cells. However, their study found that neuregulin 1(Nrg1) expression was reduced and Nrg1-mediated cardiac erb-b2 receptor tyrosine kinase 2 and 4 (ErbB2/4) signaling was impaired by knocking out YAP and TAZ in the embryonic endocardium. This affects the differentiation of trabecular muscle cells, leading to a thin, dense myocardium and early postnatal mortality.

The rapid proliferation of second cardiac field (SHF) progenitor cells occurs at the two poles of the heart to continuously extend the cardiac tube (Christoffels and Jensen, 2020). During differentiation from the mesoderm to CPCs in the heart, the Hippo pathway determines the number of SHF CPCs formed at the early cardiac venous pole, which further determines the size of the atrium. This process occurs by inhibiting the activity of YAP/TAZ and expression of *bone morphogenetic protein 2b (Bmp2b)* and *heart and neural crest derivatives expressed 2(hand2)* (Fukui et al., 2018). The epithelial tension generated in this process promotes the extension of the cardiac tube, and that mechanical tension affects the speed and direction of cell division. In the corresponding area of the embryonic epithelial tension increase, cell mitosis is also significantly increased, and the level of YAP/TAZ expression is also highly increased in the nucleus. When a YAP inhibitor is used, the proliferation of epithelial cells is significantly reduced, indicating that YAP may convert mechanical force signals into cell division and proliferation signals, thus promoting the extension of the cardiac tube (Francou et al., 2017).

#### 4.1.3 LATS1/2

The knockout of LATS1/2 in the epicardium of mouse embryos is embryonically lethal, resulting in defective coronary arteries (Singh et al., 2016; Xiao et al., 2018). Gan M. et al. (2020) reported that p-LATS1/2 inhibits mTORC1 signaling to control heart size by directly phosphorylating S606 of Raptor to attenuate mTORC1 kinase activation.

Loss of LATS1/2 promotes an increase in the number of C20 cells, a transitional cell type with the same characteristics as epicardial and primitive cardiac fibroblasts, in the subepicardial space while increasing the nuclear activity of YAP. YAP-TEAD inhibits the expression of *dipeptidyl peptidase 4*(*Dpp4*) and *dehydrogenase/reductase 3(Dhrs3)*, leading to defective differentiation of subepicardial C20 cells into fibroblasts and defective coronary vascular development (Xiao et al., 2018). Notably, phosphorylated MST1 activates the Hippo pathway via phosphorylation of LATS2, further increasing cardiomyocyte apoptosis by upregulating Bcl-xL expression. LATS2 can also decrease protein synthesis by increasing the phosphorylation of the negative regulator of hypertrophy, eEF2, by reducing the phosphorylation of Akt and p70S6K independently of regulating the apoptosis system and decreasing cardiomyocyte size *in vitro*. Mice overexpressing *LATS2* exhibited a decline in ventricular mass accompanied by limited diastolic function, and the number of single cells did not change, further demonstrating that the Hippo pathway controls the size of the heart by promoting cardiomyocyte apoptosis and inhibiting cardiomyocyte hypertrophy in mice after birth (Matsui et al., 2008).

#### 4.1.4 TEAD

TEAD has been demonstrated to interact with myocyte serum response factor (SRF) and myocyte enhancer factor-2 (MEF2) to regulate the expression of cardiomyocyte genes and is necessary for the development of heart embryos (Chen et al., 1994; Gupta et al., 2001; Maeda et al., 2002). TEAD is highly expressed in the late embryo and early postnatal periods, which coincides with the proliferation of cardiac myocytes (Liu et al., 2017).

#### 4.1.5 Mst1/2

Li et al. (Li et al., 2013) knocked out *Mst1* and *Mst2* in mice embryo cells, which resulted in fast proliferation of *Mst−/−ES* cells accompanied by a decrease in intracellular phosphorylated YAP levels and an increase in YAP nuclear activity. At the same time, the expression of the YAP downstream target genes *connective tissue growth factor* (*Ctgf*) and *cysteine rich angiogenic inducer 61* (*Cyr61*) increased; however, *Mst−/− ES* cells failed to differentiate into CPCs despite the mesoderm lineage. In addition, in *Mst−/− ES* cells, the expression of the pluripotency marker Nanog was upregulated and that of the non-canonical Wnt pathway ligands *Wnt2*, *Wnt2b,* and *Wnt5a* that enhance the differentiation of embryonic stem cells into cardiomyocytes *was* downregulated. Therefore, *Mst1/Mst2* is necessary for embryonic stem cells to differentiate into progenitor cells and cardiomyocytes.

#### 4.1.6 VGLL4

Lin et al. (Lin et al., 2016) found that VGLL4 was an important regulator of *postpartum* cardiac growth and maturation. They found that the VGLL4 levels in mouse hearts increased with age. In newborn hearts, VGLL4 cannot interact with TEAD because acetylation by acetyltransferase p300 and the YAP-TEAD interaction increases, promoting the proliferation of early myocardial cells. In the adult heart, VALL4 binds to and degrades TEAD, thereby inhibiting the proliferation of myocardial cells and promoting cell necrosis, possibly affecting postnatal cardiac maturation.

### 4.2 ncRNA/hippo pathway in cardiac development

In recent years, ncRNAs have been identified as key factors in regulating gene expression and have received widespread attention in the field of cardiac cell regeneration. Particularly, in a large-scale screening study of the mechanism by which miRNAs promote the proliferation and regeneration of cardiac myocytes, researchers found that most miRNAs promote the proliferation of cardiac myocytes through inhibition of the Hippo pathway and nuclear translocation and activation of YAP, which are crucial for cardiomyocyte proliferation and cardiac development (Diez-Cuñado et al., 2018; Braga et al., 2021) (Table 2).

**TABLE 2:** ncRNAs/Hippo pathway in cardiac development.

ncRNA	Model	Pattern	Hippo targets	Results	Ref.
MiR-520d-3p	HiPSC-CM (miRSystem software predict)	Direct target	LATS2 and TEAD1	Promote proliferation	Diez-Cuñado et al. (2018)
miR-590-3p	HiPSC-CM (miRSystem software predict)	Direct target	YAP and TEAD	Promote proliferation	Diez-Cuñado et al. (2018)
miR-17	HiPSC-CM (miRSystem software predict)	Direct target	MST2, SAV1, LATS2, MOB1A, TEAD1, and TEAD3	Promote proliferation	Diez-Cuñado et al. (2018)
miR302-367	Mice	Direct target	LATS2↓, Mob1b↓, and MST1↓	Promote proliferation	Tian et al. (2015)
miR-199a-3p, miR-1825, miR-302d, and miR-373	Rat	Targets Cofilin2	Nuclear YAP↑	Promote proliferation	Torrini et al. (2019)
miR-199a-3p	Rat	Targets TAOK1 and b-TrCP	Nuclear YAP↑	Promote proliferation	Torrini et al. (2019)
MicroRNA-302d	hPSC-CMs	Direct target	LATS2↓→nuclear YAP↑	Promote proliferation	Torrini et al. (2019)
MircroRNA-10b	hESC-CMs	Direct target	LATS1↓→nuclear YAP↑	Promote proliferation	Xie et al. (2020)
miR-540-3p	Mice/ESC CMs	Directly target Dtna (key component of DGC) and hinder the DGC-YAP interaction	Nuclear YAP↑	Promote the proliferation of cardiomyocytes and hinder their maturation	Wu et al. (2022)
lncRNA -Cmarr	Mice/ESC CMs	Inhibit the effect of miR-540-3p competitively	Nuclear YAP↓	Promote the transition of cardiomyocytes from proliferation to maturation	Wu et al. (2022)
*Platr4* (lncRNA)	Mice ESC	Molecular scaffold or chaperone	YAP -TEAD4↑	Cardiogenic mesodermal lineage differentiation	Hazra et al. (2022)
*BANCR*	Primate pluripotent stem-cell-derived cardiomyocytes	TEAD4 binds with BANCR’s enhancer	Downstream target of YAP-TEAD4	Promote cardiomyocyte migration	Wilson et al. (2020)
LncDACH1	Mice	Binds to PP1A	YAP↓	Inhibit myocardial cell proliferation associated with *postpartum* cardiac arrest of proliferation	Cai et al. (2020)

#### 4.2.1 miR-520d-3p/miR-590-3p/miR-17

In a large-scale analysis of miRNAs, researchers screened 67 miRNAs that potentially act on the Hippo pathway, activate YAP nuclear translocation, and maintain DNA synthesis and cell division in immature cardiomyocytes, thereby promoting iPSC-derived cardiomyocyte proliferation. In their prediction model, each miRNA acted on multiple Hippo pathway targets. For example, the predicted targets of miR-520d-3p are LATS2 and TEAD1, those of miR-590-3p are YAP and TEAD, and the predicted targets of miR-17 family members can inhibit the MST2, SAV1, LATS2, MOB1A, TEAD1, and TEAD3 Hippo pathway molecules (Diez-Cuñado et al., 2018).

#### 4.2.2 miR-302-367

Tian et al. (Tian et al., 2015) showed that miR-302-367 clusters are important for the regulation of embryo cardiac development because cardiac cells show abnormal phenotypes, such as ventricular wall thinning, abnormal ventricular septal development, and reduced cardiomyocyte proliferation, after the knockout of these proliferative miRNAs. In the model overexpression of miR302-367, cardiac ejection function was limited, cardiac function was poor, cardiomyocyte proliferation increased, accompanied by a significant increase in the percentage of mononuclear and dual-core myocytes, and the volume of these cells was significantly reduced, indicating that overexpression of miR302-367 promotes cardiomyocyte proliferation but affects cardiomyocyte and cardiac maturation. Additionally, miR302-367 was only expressed in embryonic mice and directly targeted the mRNA of LATS2, Mob1b, and MST1 to inhibit hippo pathway (Tian et al., 2015).

#### 4.2.3 miR-199a-3p/miR-1825/miR-302d/miR-373

miR-199a-3p, miR-1825, miR-302d, and miR-373 directly target Cofilin2, which inhibits the depolymerization of filamentous actin, resulting in YAP nuclear translocation accompanied by activation of the expression of YAP target genes *CTGF* and *CyR6*, illustrating the critical role of the correct formation of the cytoskeleton in mediating mechanical signals to activate the YAP pathway (Torrini et al., 2019). In the same study, miR-199a-3p was found to promote cardiomyocyte proliferation by directly targeting the mRNA of the upstream activator TAOK1 of the Hippo pathway and E3 ubiquitin ligase (β-TrCP), which mediates the degradation of YAP via ubiquitination, thus inhibiting the Hippo pathway and promoting YAP nuclear translocation (Torrini et al., 2019). However, the above experiments were based on rat cells owing to the poorly conserved functions of miRNAs among different species (Diez-Cuñado et al., 2018).

#### 4.2.4 miR-302d/miR-10b

Xu et al. studied miRNA Hippo pathway targets in human pluripotent stem cell-derived CMs (hPSC-CMs) and hESC-CMs and found that miR-302d and miR-10b promote cardiomyocyte proliferation and have no significant effect on CM maturation, by directly targeting *LATS2* and *LATS1* in the Hippo pathway, respectively. Simultaneously, the inhibition of LATS2 was accompanied by enhanced YAP nuclear activity and significant upregulation of its target genes, *amphiregulin* (*AREG*), *CTGF,* and *C-X-C motif chemokine ligand 5(CXCL5)*. As the proliferation of hPSC-CMs and hESC-CMs is similar to that of cardiomyocytes during mammalian embryonic development, this largely simulates the regulation of the Hippo pathway by ncRNA in cardiomyocytes during mammalian heart development. Moreover, miR-302d and miR-10b are enriched early in hPSC-CMs and hESC-CMs, and their expression levels decrease with embryonic development, which is consistent with that in the development and maturation of the heart (Xu et al., 2019; Xie et al., 2020).

#### 4.2.5 miR-540-3p/lncRNA-cmarr

As previously mentioned, in cardiomyocytes, the DGC binds to YAP and blocks its transfer from the cytoplasm to the nucleus, thereby promoting the maturation transition of cardiomyocytes. Therefore, the interaction between the Hippo pathway and DGC controls the transition of cardiomyocytes from proliferation to maturation. However, miR-540-3p directly inhibits the expression of Dtna (a key component of the DGC), blocks the DGC-YAP interaction, and increases the percentage of nuclear YAP, leading to the expression of YAP downstream target genes, thereby promoting the proliferation of cardiomyocytes and inhibiting their maturation (Wu et al., 2022).

Cmarr, which is upregulated in cardiomyocytes during heart development, promotes structural and physiological maturation of mouse embryonic stem cell CMs (mESC CMs). As cardiac maturity increases, Cmarr expression is further upregulated and improves the electrophysiological properties, calcium-handling capacity, and mitochondrial content of cardiomyocytes, thereby increasing cardiac contractile function in mature hearts. Further research revealed that Cmarr, as a competitive endogenous RNA, blocks the inhibition of *dystrobrevin alpha* (*Dtna*) expression by miR-540-3p and promotes the interaction between the DGC and YAP, thereby reducing the proportion of nuclear YAP and the expression of YAP target genes and promoting the transition from proliferation to maturation of cardiomyocytes. Therefore, the Cmarr/miR-540-3p axis promotes cardiomyocyte maturation transition by coordinating the expression of *Dtna* (Wu et al., 2022).

#### 4.2.6 lncRNA Platr4

A recent study found that *Platr4*, a lncRNA specifically expressed in embryonic stem cells and early developing embryos, specifically combines with YAP and TEAD4 in the form of a molecular scaffold or chaperone to increase the interaction of YAP-TEAD4, further promoting the expression of *connective tissue growth factor* (*Ctgf*), also known as *cellular communication network 2 (Ccn2),* a candidate extracellular matrix (ECM) protein downstream of YAP-TEAD, which is required for fetal cardiogenic mesoderm differentiation. They also found that the loss of Platr4 caused increased ventricular wall thickness, limited cardiac contractile function, and decreased cardiomyocyte contractile function, which is related to reduced expression levels of cardiac troponin T (cTnT) and myosin heavy chain 7b (myh7b) (Hazra et al., 2022).

#### 4.2.7 lncRNA BANCR

The lncRNA *BANCR*, which exists only in primate fetal cardiac myocytes, was found to be specifically bound by TEAD4 and plays a role in regulating the migration of cardiac myocytes as a downstream target of YAP/TEAD. YAP knockout resulted in a significant decrease in BANCR expression in hESC-CMs. At the same time, this study found that all four exons in BANCR have the biological function of promoting radial cell migration. By constructing a BANCR-knockout mouse model, researchers found that the heart size of mice increased but was not accompanied by an increase in cardiomyocyte proliferation; this phenomenon requires further research (Wilson et al., 2020).

#### 4.2.8 LncRNA DACH1

The lncRNA DACH1 was found to be significantly upregulated during *postpartum* cardiac development. In specific LncDACH1 transgenic (TG) mice, myocardial cell proliferation and mitosis were inhibited by promoting the phosphorylation and cytoplasmic retention of YAP. The decrease in YAP activity was accompanied by a decrease in the expression of the YAP target gene, *Ctgf,* which binds to protein phosphatase 1 catalytic subunit alpha (PP1A). Thus, LncDACH1 is associated with the loss of postnatal myocardial cell regeneration ability (Cai et al., 2020).

## 5 Non-coding RNA/Hippo pathway in cardiac diseases

### 5.1 Heart valve development

The valves of the vertebrate heart include the atrioventricular (AV) valves (the mitral and tricuspid valves) and semilunar SL valves (the aortic and pulmonary valves). In recent years, a large number of studies have proved that the BMP/TGFβ, Wnt/β-catenin, Hippo/Yap, Notch, VEGF, NFATc1, Has2/ErbB2, ErbB3/Ras, and NF1/Ras signaling pathways play key regulatory roles in the endothelial-mesenchymal transition (EMT) and the formation of heart valves (Armstrong and Bischoff, 2004; O'Donnell and Yutzey, 2020). However, there is currently limited research on the role of the Hippo pathway in heart valve formation.

#### 5.1.1 Let-7/miR-16/miR-21/miR-23a/miR-29/miR-107/miR-152

Nuclear YAP/TAZ may stimulate the biogenesis of miR-16, miR-21, miR-23a, miR-29, miR-107, and miR-152 by increasing the activity of DICER and inhibiting the accumulation of miR-let7, which links the Hippo pathway to miRNA biogenesis (Tumaneng et al., 2012; Chaulk et al., 2014). YAP/TAZ senses that low cell density, its nuclear translocation increases, which increases the level of LIN28 through post-transcriptional regulation. LIN28 regulates the production of miR-let-7 by recognizing the Let-7 pre-miRNA hairpin, thereby inhibiting the accumulation of let-7, promoting the activity of DICER, stimulating the processing of miRNA, increasing the production of miRNA, and thus may be involved in the regulation of genes related to valve development (Chaulk et al., 2014).

#### 5.1.2 miR-98/miR-29/miR-30

Loss of DICER may cause mitral valve malformations during embryonic development, leading to congenital mitral regurgitation and stenosis. Yan et al. (Yan et al., 2022) found that DICER activity is only essential for mitral valve development; accordingly, miRNAs also differentially mediate gene regulation in the two AV valves. DICER is required for the expression of the ECM genes Col1a1, Col3a1, Flna, and Tnxb, which encode many ECM components. Col1a1 and Col3a1 are directly regulated by miR-98 and miR-29, whereas *tenascin XB (Tnxb)* is regulated by miR-30 in mitral valve endocardium-derived mesenchymal cells. Inactivation of DICER leads to the downregulated expression of the above miRNAs, resulting in an increase in the corresponding ECM genes, which leads to hyperplastic and abnormally shaped leaflets of the mitral valves by inducing cell condensation (Yan et al., 2022). The above findings suggest that the Hippo pathway participates in the synthesis of miRNAs by affecting DICER activity and plays a crucial role in the correct formation of embryonic heart valves. However, this requires further investigation.

#### 5.1.3 lncRNA Platr4

A recent study showed that the lncRNA Platr4, which can bind to YAP and TEAD, is specifically expressed in early embryonic development and mature heart valves and its knock down in mice results in valvular defects with fibro osseous metaplasia, fibrocartilaginous metaplasia, and valve mucus degeneration, accompanied by a decrease in the ECM-related protein CTGF, indicating that Platr4 may play a role in the formation of the structure and components of the valve ECM and the development and stable maintenance of mature valves, but there is currently no relevant research on Platr4 in the development of embryonic valves (Hazra et al., 2022).

Even though there is no direct evidence to prove that ncRNA plays a role in the regulation of valve formation via the Hippo pathway, the above findings suggest that ncRNA plays a role in this process, but further research is needed.

### 5.2 Cardiomyopathy

During the occurrence and development of cardiomyopathy, some ncRNAs regulate the progression of the disease by regulating the Hippo pathway. The current findings are primarily concentrated on the regulation of cardiac hypertrophy, fibrosis, and apoptosis.

#### 5.2.1 miR-21-5p/miR-135b

In an analysis of differentially expressed miRNAs from 24 histologically confirmed arrhythmogenic cardiomyopathy (ACM) hearts, researchers identified 21 miRNAs that were differentially expressed compared to those in the control group, including miR-21-5p and miR-135b, which are known to regulate the Hippo signaling pathway in cancer cells. Their increased expression was accompanied by an increase in the expression of the target genes *bone morphogenetic protein receptor type 2(BMPR2)* and *transforming growth factor beta receptor 2(TGFBR2)*, which are related to adipogenesis and extracellular matrix generation. Therefore, they may also play a role in Hippo signaling in fat generation in ACM; however, further relevant experimental evidence is needed to prove this (Zhang et al., 2016).

#### 5.2.2 miR-550a-3-5p/miR-27b-3p/miR-195-5p/miR-9-3p/miR-103a-3/miR-429

Piquer-Gil et al. (Piquer-Gil et al., 2022) identified several molecules that may play a role in ACM by analyzing ncRNAs that act on Hippo pathway molecules in tumors that require further basic experiments for confirmation. Among them, some ncRNAs can participate in the pathogenesis of tumors by regulating gene expression via the Hippo pathway; for example, miR-550a-3-5p, miR-27b-3p, miR-195-5p, and miR-9-3p may be involved in the occurrence of ACM by directly inhibiting the activity of YAP, while miR-103a-3p and miR-429 can reduce the phosphorylation of YAP by inhibiting the phosphorylation of LATS2 and enhancing the nuclear activity of YAP.

#### 5.2.3 LncRNA UCA1/miR-18a, lncRNA FLVCR1-AS1/miR-513, and lncRNA RP11-323N12.5

Piquer-Gil et al. (Piquer-Gil et al., 2022) also reported that axes such as lncRNA UCA1/miR-18a/YAP and lncRNA FLVCR1-AS1/miR-513/YAP promote YAP activity through miRNA inhibition. In addition, there may be a bidirectional relationship between YAP/TAZ and ncRNA. For example, YAP and lncRNA RP11-323N12.5 can bidirectionally regulate the activities of both genes, thereby promoting the expression of genes regulated by YAP. However, the role of these targets in heart disease need to be further investigated (Piquer-Gil et al., 2022).

#### 5.2.4 miR-206

miR-206 expression is downregulated by MST1 and upregulated by YAP, and its expression increases mRNA expression of the hypertrophic marker gene 24ND, and targets and degrades tumor suppressor forkhead box protein P1 (FoxP1) to mediate cardiomyocyte hypertrophy and survival induced by YAP. However, this was considered as physiological hypertrophy that can play a role in protecting myocardial damage from higher pressure loads and I/R damage; however, it is unknown whether miR-206 plays a role in hypertrophic cardiomyopathy (Yang et al., 2015).

#### 5.2.5 LncRNA MALAT1

Liu et al. constructed a mouse model of diabetic cardiomyopathy using si-MALAT1 and found that silencing MALAT1 can reduce the phosphorylation of MST1 and LATS1 in high-glucose-induced cardiac fibroblasts and promote YAP nuclear translocation, thereby reducing the accumulation of inflammation-related factors TNF-α, IL-1β and collagen (type I and type III) under a high-glucose environment, thereby reducing the inflammatory response and myocardial fibrosis. The lncRNA MALAT1 is upregulated in a high-glucose environment and regulates YAP nuclear translocation by binding to CREB to promote myocardial fibrosis by promoting cardiac fibroblast proliferation and collagen expression, thus aggravating interstitial fibrosis and myocardial damage in diabetic cardiomyopathy (Liu et al., 2020).

#### 5.2.6 Circ-CDR1as

In addition, the expression of circular RNA cerebellar degeneration-related protein 1 antisense (Circ-CDR1as) is also upregulated in diabetic cardiomyopathy, which activates the Hippo pathway involved in the function of Alk B homolog 5 (ALKBH5), an m6A demethylation enzyme, and FOXO3 by significantly inhibiting mammalian sterile 20-like kinase 1 (MST1) ubiquitination degradation, thereby mediating cardiomyocyte apoptosis. Further research has shown that defects in Circ-CDR1as can reduce the rate of cardiomyocyte apoptosis, and the cardiac function of DCM mice was significantly improved in a Circ-CDR1as knockout mouse model (Shao et al., 2022).

### 5.3 Heart failure

Currently, there are few studies on the involvement of ncRNA in heart failure (HF) via the regulation of the Hippo pathway. A KEGG pathway annotation study showed that several miRNAs differentially expressed between cardiac hypertrophy rats and healthy rats, such as let-7e-5p, miR-328a-3p, miR-21-5p, miR-222-3p, miR31a-5p, miR-423-5p, miR-144-3p, miR-451-5p, miR-3068-5p, miR-142-3p, miR-26b-5p, and miR-133b-3p, are likely concentrated in the Hippo pathway (Gan et al., 2020). Among them, the expression of miR-133b was found to be downregulated in a MHCα-CN mouse heart failure model [85], while the expression of let-7e-5p, miR-21-5p, and miR-451 was upregulated in heart failure patients [86]. However, whether these ncRNAs specifically function via the Hippo pathway remains unknown. Furthermore, although YAP/TAZ can promote the expression of fibrosis-promoting genes, such as CTGF and PDGF, there are currently no studies on the regulation of TAZ by ncRNAs in heart failure to promote myocardial fibrosis (Ghafouri-Fard et al., 2021).

#### 5.3.1 circRNAYap

The level of the YAP cyclic RNA isomer circRNAYap, produced by partial reverse splicing of the YAP gene exon, is decreased in stress overload mouse models. Further studies have found that increased expression of circRNAYap can be mediated by association with tropomyosin 4, and γ- Actin binding reduces actin aggregation to alleviate myocardial fibrosis, thereby improving cardiac function (Wu et al., 2021).

#### 5.3.2 LncExACT1

The expression of long non-coding exercise-associated cardiac transcript1 (LncExACT1), a long chain ncRNA closely related to exercise, is upregulated in heart failure, and downregulated in exercised hearts. Inhibition of LncExACT1 in the exercising heart can increase physiological hypertrophy and cardiomyocyte proliferation and can also prevent myocardial fibrosis and dysfunction, which can protect the heart from adverse remodeling caused by TAC, thereby delaying and preventing heart failure. In contrast, overexpression of lncExACT1 can cause cardiac hypertrophy and fibroblast proliferation and participates in the occurrence and development of heart failure. Further studies have shown that lncExACT1 regulates Hippo/YAP1 signaling through DCHS2. lncExACT1-DCHS2 increases the content of p-YAP, inhibits the nuclear and transcriptional activity of YAP1, inhibits the role of YAP1 in exercise-induced cardiac growth and myocardial production, and plays a role in promoting pathological myocardial hypertrophy and fibrosis, thereby promoting the development of cardiac dysfunction (Li et al., 2022a).

### 5.4 Coronary heart disease

The formation of foam cells is a hallmark of atherosclerosis. A study found 84 differentially expressed miRNAs implicated in the mechanism by which macrophages engulf and clear excess ox-LDL accumulated in the neointima to form foam cells. KEGG pathway analysis showed that some of these miRNAs converged with the Hippo pathway, which may be involved in the transport and metabolism of ox-LDL, formation of foam cells, and the apoptosis and inflammatory responses of macrophages (Li et al., 2018).

#### 5.4.1 MiR-200c-3p

A study by Mao et al. showed that miR-200c-3p was highly expressed in an atherosclerosis (AS) mouse model established by feeding ApoE−\/- mice with a high-fat diet, and promoted the process of ox-LDL induced EMT in human umbilical vein endothelial cells (HUVECs) by suppressing the SMAD7/YAP pathway, thereby promoting the transformation of ox-LDL-treated HUVECs from a cobblestone-like epithelial phenotype to a spindle-like mesenchymal phenotype, which may be involved in atherosclerosis (Mao and Jiang, 2021).

#### 5.4.2 MiR-496

Notably, other studies have shown that ox-LDL stimulation upregulates the expression of miR-496, inhibits the expression of YAP protein, and reduces the nuclear translocation of YAP, leading to the loss of downstream gene expression, increased endothelial cell apoptosis, reduced vascular endothelial cell proliferation and migration, and vascular endothelial cell dysfunction (Hu et al., 2019).

### 5.5 Myocardial infarct

Some ncRNAs can promote the regeneration of myocardial cells by regulating YAP, providing a promising therapeutic strategy for the recovery of cardiac function after myocardial infarction.

#### 5.5.1 MiR-93

Through *in vivo* and *in vitro* experiments, Ma et al. found that miRNA-93 significantly reduced LATS2 expression and YAP phosphorylation in the myocardium after myocardial infarction by targeting LATS2, which inactivated the Hippo/YAP pathway, increased YAP nuclear activity and transcriptional activity, inhibited myocardial fibrosis, and promoted cardiomyocyte viability, thereby improving cardiac function preservation after myocardial infarction. In addition, upregulated expression of miR-93 can increase endothelial cell viability and migration by inhibiting the Hippo pathway, promoting angiogenesis in the ischemic heart, inhibiting myocardial cell apoptosis, reducing infarct size, and exerting a protective effect on myocardial infarction, thereby leading to the improvement of post-MI cardiac function. In contrast, overexpression of miR-93 reduced the expression of matrix metalloproteinases (MMPs) involved in collagen deposition by inhibiting the phosphorylation of LATS2, reducing collagen deposition, inhibiting fibrosis, and alleviating remodeling. The upregulation of miR-93 expression can also reduce the expression levels of the pro-apoptotic proteins cleaved caspase-3 and Bax and increase the expression levels of the anti-apoptotic protein Bcl2 by inhibiting the Hippo pathway, thereby protecting endothelial cells from hypoxia/reoxygenation damage and subsequent apoptosis. In summary, miR-93 directly inhibits the Hippo pathway activity by targeting LATS2, thereby improving cardiac function after myocardial infarction (Ma et al., 2020).

#### 5.5.2 miR302-367

Overexpression of miR302-367 can promote cardiomyocyte proliferation and improve the prognosis of myocardial infarction by inhibiting Mst1 and Mob1b activity in the Hippo pathway. In mouse models overexpressing miR302-367, they found that the expression of phosphorylated YAP was reduced; however, the expression of nuclear YAP was enhanced, and the cell cycle of postnatal cardiomyocytes was reactivated. Experimental results showed that overexpression of miR302-367 also increased the number of cardiomyocytes undergoing mitosis (PH3+) and cytokinesis (Aurora B kinase+). In contrast, inhibition of the Hippo pathway was accompanied by a decrease in the expression of genes related to programmed cell death in cardiomyocytes. Simultaneously, an increase in cardiomyocytes leads to reduced fibrosis and scarring after myocardial infarction. Therefore, increased miR302-367 levels can improve the prognosis of myocardial infarction by promoting cardiomyocyte proliferation (Tian et al., 2015).

However, overexpression of miR302-367 is not advantageous. Sustained overexpression of miR302-367 can lead to the dedifferentiation and dysfunction of cardiomyocytes. Moreover, studies have shown that sustained overexpression of miR302-367 for 3 weeks after myocardial infarction can lead to ventricular dilation and reduced ejection fraction, accompanied by continued upregulation of the cell proliferation marker Cks2, as well as continued downregulation of the ratio of Myh6 (α-myosin heavy chain) to Myh7 (α-myosin light chain), which correlates with sarcomere division and continued dedifferentiation of cells. Tian et al. designed a simulated substance that can be instantaneously expressed in the myocardium to perform cardiac repair and improve cardiac function within a certain range and degree. Therefore, this may be a promising treatment method but requires further research (Tian et al., 2015).

#### 5.5.3 miR-411

Overexpression of miR-411 has also been found to inhibit the phosphorylation of YAP and increase its nuclear activity by inhibiting the phosphorylation of LATS1 in the Hippo pathway, increasing the proliferation and survival of cardiomyocytes, and improving cardiac phenotypes after myocardial infarction in mice. Studies have found that the expression level of miR-411 in NRCMs is higher than that in neonatal rat cardiac fibroblasts (NRCFs), and the expression levels of YAP target genes (such as *baculoviral IAP repeat containing 5(Birc5)*, *fibroblast growth factor 2(Fgf2)*, and TEAD1) were increased in cardiomyocytes transfected with miR-411, which also exhibited higher levels of the cell cycle markers Ki-67 and phosphohistone H3 (pHH3) and EdU incorporation, indicating that increased mitosis and DNA synthesis can induce adult cardiomyocytes to re-enter the cell cycle and improve viability (Nugroho et al., 2022).

#### 5.5.4 lncRNA-cmarr

lncRNA-cmarr, which is highly expressed in the heart and may be related to heart maturation, has been shown to act as a competitive ncRNA to block the inhibition of miRNA-540-3p on Dtna expression, promote binding of the dystrophin glycoprotein complex (DGC) and YAP, and reduce the proportion of nuclear YAP and the expression of YAP target genes. At the same time, in the hearts of myocardial infarction mice overexpressing Cmarr, Cmarr was found to promote the maturation of cardiomyocytes derived from embryonic stem cells, reduce infarct size, and increase the ejection fraction by enhancing blood vessel density in the host heart, thus improving cardiac function after myocardial infarction (Wu et al., 2022).

### 5.6 Arrhythmias

Similarly, KEGG pathway analysis revealed that a series of differentially expressed miRNAs concentrated in the Hippo pathway may be helpful in protecting against ventricular arrhythmias caused by pressure overload. However, further research is needed (Xu et al., 2018).

### 5.7 Cardio-oncology

#### 5.7.1 let-7i

A study on extracellular vesicles found that let-7i alleviated DOX-induced cardiotoxicity, prevented heart damage, and exerted a cardioprotective effect by inhibiting YAP activity. Extracellular vesicles derived from trophoblastic stem cells (TSCs) could transfer miRlet-7i to myocardial cells, inhibit the inflammatory response, and reduce myocardial cell apoptosis and fibrosis by downregulating YAP1 activity, accompanied by a decrease in the expression levels of the YAP target genes *ctgf* and *TEAD*. Thus, DOX-induced myocardial remodeling and heart failure can ultimately be reversed, thereby improving dilated cardiomyopathy and cardiac dysfunction caused by doxorubicin treatment (Ni et al., 2020).

#### 5.7.2 miR-125b

miR-125b is upregulated in cells treated with doxorubicin and may serve as a potential biomarker of DOX-induced cardiotoxicity. The upregulated expression of miR125b was accompanied by a decrease in the levels of DOX-induced oxidative stress markers (catalase (CAT), superoxide dismutase (SOD), glutathione (GSH), and glutathione peroxidase (GSH-Px)) and an increase in malondialdehyde (MDA), indicating that miR125b may mediate DOX-induced cardiotoxicity produced by DOX through oxidative stress. Furthermore, miR-125b has also been shown to target *StAR related lipid transfer domain containing 13(STARD13)*, inhibit YAP phosphorylation and nucleocytoplasmic translocation, enhance YAP transcriptional activity, and promote doxorubicin-induced cardiotoxicity by promoting cardiomyocyte apoptosis and reducing cell viability. Similarly, miR-125b inhibition is accompanied by a reduction in ROS levels and cardiomyocyte apoptosis, increases cardiomyocyte activity, and exerts a cardioprotective effect. However, the underlying mechanisms require further research (Jin et al., 2022).

#### 5.7.3 miR-4732-3p

Sánchez-Sánchez et al. studied the downregulated expression of miR-4732-3p and its possible effect on the Hippo pathway by analyzing differentially expressed miRNAs and their predicted targets in cardiotoxicity induced by doxorubicin. Upregulated expression of miR-4732-3p can protect cells from oxidative stress, prevent cell apoptosis, significantly increase angiogenesis, and inhibit myocardial fibrosis. Therefore, they hypothesized that miR-4732-3p might promote the nuclear translocation of YAP by inhibiting its phosphorylation, thereby improving cardiac dysfunction caused by doxorubicin toxicity; however, further systematic research is required (Sánchez-Sánchez et al., 2022) (Table 3).

**TABLE 3:** Main studies in the cardiology field regarding the Hippo signaling pathway and ncRNAs.

Cardiovascular diseases	Model/species	ncRNA	Hippo core mechanism	Results	Ref.
Heart valve development	MCF10A cells/mice	miR-let7↓→Dicer↑→miR-16, miR-21, miR-23a, miR-29, miR-107 and miR-152↑	May be stimulated by YAP/TAZ	Ensure correct formation of embryonic heart valves	Tumaneng et al. (2012), Chaulk et al. (2014), Yan et al. (2022)
	Mouse/embryonic cardiomyocytes	DICER↓→miR-98, miR-29, miR-30↓	May be stimulated by YAP/TAZ	Lead to excessive proliferation of the extracellular matrix, producing an abnormal valve phenotype	Yan et al. (2022)
	Mice	LncRNA Platr4↓	YAP -TEAD4↓	Valvular defects with fibrocartilaginous metaplasia, fibro osseous metaplasia, and myxoid degeneration	Hazra et al. (2022)
Arrhythmogenic cardiomyopathy (ACM)	Human	miR-21-5p and miR-135b	Hippo?	Promote extracellular matrix and lipogenesis	Zhang et al. (2016)
	Hypothesis	miR-550a-3-5p/miR-27b-3p/miR-195-5p/miR-9-3p/	YAP↓	May be related to the pathogenesis of ACM	Piquer-Gil et al. (2022)
	Hypothesis	miR-103a-3/miR-429	p-LATS2↓→p-YAP↓→nuclear YAP↑	May be related to the pathogenesis of ACM	Piquer-Gil et al. (2022)
	Hypothesis	LncRNA UCA1/miR-18a, lncRNA FLVCR1-AS1/miR-513, lncRNA RP11-323N12.5	YAP↑	May be involved in the pathological process of ACM by promoting the expression of YAP target genes	Piquer-Gil et al. (2022)
Cardiac hypertrophy	Mice/neonatal rats CMs	MiR-206↑	Downstream target of YAP	Promote myocardial hypertrophy and survival	Yang et al. (2015)
Diabetic cardiomyopathy	Mice/neonatal mouse cardiac fibroblasts (CFs)	LncRNA MALAT1↓	p-MST1↓→p- LATS1↓→nuclear YAP↑	Aggravate interstitial fibrosis and myocardial damage in diabetic cardiomyopathy	Liu et al. (2020)
	Mouse/cardiomyocytes	Circ CDR1ase	MST1↑	Promote apoptosis and myocardial damage	Shao et al. (2022)
Heart failure	Mouse/cardiomyocytes	circRNA Yap	YAP circular RNA isoform produced by partial back splicing of YAP gene exon	Reduce myocardial fibrosis, thereby improving cardiac function	Wu et al. (2021)
	Mouse cardiomyocytes/zebrafish	Lnc ExACT1	p-YAP↑→nuclear YAP↓	Cardiac rational hypertrophy and fibroblast proliferation participate in the occurrence and development of heart failure	Li et al. (2022a)
Coronary heart disease	Mice/HUVECs	miR-200c-3p	YAP↓	Promote ox-LDL-induced endothelial to mesenchymal transition	Mao and Jiang (2021)
	Mice/HUVECs	miR-496	Nuclear YAP↓	Induce vascular endothelial cell dysfunction and atherosclerosis	Hu et al. (2019)
Myocardial infarct	Mice/HUVECs	miR-93	LATS2↓→p-YAP↓→nuclear YAP↑	Inhibit myocardium remodeling and improve cardiac function	Ma et al. (2020)
	Mouse/cardiomyocytes	miR302-367	MST1, Mob1b↓→ p-YAP↓→nuclear YAP↑	Promote myocardial regeneration and reduce fibrotic scars	Tian et al. (2015)
	RAT/NRCMs	miR-411	p-LAST1↓→ p-YAP↓→nuclear YAP↑	Inhibit cardiomyocyte apoptosis, promote myocyte proliferation	Nugroho et al. (2022)
	mice/mESC-CM	lncRNA-cmarr/miRNA-540-3p	Nuclear YAP↓	Promote the maturation of cardiomyocytes, thereby improving cardiac function after myocardial infarction	Wu et al. (2022)
Cardio-oncology	Mice/AC16cells	Let-7i	YAP↓	Inhibit apoptosis and fibroblast and reverse DOX-induced myocardial remodeling and heart failure	Ni et al. (2020)
	Mice/H9C2 cells	miR-125b	p-YAP↓→nuclear YAP↑	Promote oxidative stress and cardiotoxicity induced by doxorubicin	Jin et al. (2022)
	Computer target gene pathway prediction analysis	miR-4732-3p	p-YAP↓→nuclear YAP↑	Inhibits oxidative stress, apoptosis, and myocardial fibrosis; promotes angiogenesis	Sánchez-Sánchez et al. (2022)

## 6 Clinical applications and prospects

In the field of cardiovascular disease, current research has identified some drugs and compounds that act on the Hippo pathway and can be used for myocardial cell proliferation and regeneration, and prevention and treatment of atherosclerosis, myocardial ischemia-reperfusion injury, and heart failure. Statins, which have been widely used in clinical practice, inhibit inflammation and proliferation of endothelial cells by inactivating YAP/TAZ, thus playing an anti-atherosclerotic role (Wang et al., 2016). Curaxin CBL0137, a structural analog of 9-aminoacridine, is another small-molecule YAP inhibitor that plays the same role and is expected to become a new drug for the treatment of atherosclerosis (Ding et al., 2023). Melatonin has been found to upregulate YAP activity, promote optic atrophy 1(OPA1) related mitochondrial fusion, alleviate mitochondrial damage and myocardial cell apoptosis during ischemia-reperfusion injury, and demonstrates potential therapeutic effects (Ma and Dong, 2019).

In a study of related molecular compounds, researchers found that TT-10 increased the proliferation of myocardial cells and improved cardiac function after myocardial infarction in animals, by activating YAP nuclear activity and increasing YAP-TEAD activity (Hara et al., 2018). TRUL1 is an ATP-competitive inhibitor of LATS kinase that directly inhibits the activity of LATS, activates YAP, thereby promoting myocardial cell proliferation (Kastan et al., 2021). Another inhibitor of the Hippo pathway, XMU-MP-1, directly inhibits the kinase activity of MST1/2, enhances YAP activity, and inhibits myocardial cell apoptosis, hypertrophy, and adverse cardiac remodeling under cardiac pressure overload, thereby preventing or delaying the occurrence of heart failure (Triastuti et al., 2019).

The Hippo pathway has been widely studied as a target for tumor treatment. At present, it is known that the anticancer effects of gallic acid, icotinib hydrochloride, curcumin, ginsenoside Rg3, cryptotanshinone, nitidine chloride, cucurbitacin E, erlotinib, doxorubicin, sophoridine, cisplatin, and verteporfin are mediated by regulation of the Hippo pathway (Ghafouri-Fard et al., 2023). However, these drugs promote tumor cell apoptosis by damaging mitochondrial function, inhibiting cell viability, and causing cardiotoxicity (She et al., 2023). Additionally, due to the fact that the kinase in the Hippo pathway is a tumor suppressor factor, the upregulation of YAP activity can induce the occurrence of tumors in the body. In contrast, inhibiting the Hippo pathway and promoting YAP nuclear activity may induce regeneration of infarcted myocardial cells. Therefore, when targeting the Hippo pathway to treat heart disease, it is necessary to consider specific targeting of the heart, which involves drug carriers, administration methods, and specific durations of action.

In recent years, various carriers, including exosomes, nanomaterials, and polymers, have been used as drug carriers to accurately locate target organs in experimental and clinical research. These carriers can control the action time of drugs *in vivo*. Combining these carriers with the Hippo pathway and ncRNA may lead to a new method for the treatment of heart diseases. The use of a new catheter technique by Liu et al. to inject viral vectors into specific myocardial regions has been proven to be an effective way to activate the Hippo pathway and YAP without the occurrence of tumors in the liver and lung regions. This may serve as a novel targeted mechanism for recovery of cardiac function after myocardial infarction (Liu et al., 2021). Similarly, another research team constructed mesoporous silica nanoparticles (MSN) carrying CD11b antibodies targeting inflammatory cells as carriers of myocardial infarction. NGR1 can be specifically delivered to the heart at the infarct site through intravenous injection in a non-invasive manner, thereby promoting YAP nuclear translocation, inhibiting myocardial cell apoptosis, and controlling the inflammatory response. Meanwhile, as carriers for drug delivery, the structural advantages of MSN not only allow the effective loading and release of drugs, but also prevent cytotoxicity, demonstrating great advantages in terms of bioavailability and excretion (Li et al., 2022b). These findings provide new insights for the future development of drugs targeting the Hippo pathway components and their mechanisms of action.

The role of the Hippo pathway in regulating cardiovascular development and disease occurrence and prevention has received widespread attention. ncRNA, with its operability, detectability, specific targeting, and physiological mechanisms, can be used as a new drug to target the Hippo pathway, providing a new idea for the treatment of cardiovascular disease. However, there is still a long way to go before ncRNA can be used in clinical applications, such as the improvement of carriers, reduction of immune recognition, and missed target effects. Therefore, further studies are needed to increase the number of optimal strategies.

## References

[B1] Ali S. A. Peffers M. J. Ormseth M. J. Jurisica I. Kapoor M. (2021). The non-coding RNA interactome in joint health and disease. Nat. Rev. Rheumatol. 17 (11), 692–705. 10.1038/s41584-021-00687-y 34588660

[B2] Andergassen D. Rinn J. L. (2022). From genotype to phenotype: genetics of mammalian long non-coding RNAs *in vivo* . Nat. Rev. Genet. 23 (4), 229–243. 10.1038/s41576-021-00427-8 34837040

[B3] Armstrong E. J. Bischoff J. (2004). Heart valve development: endothelial cell signaling and differentiation. Circ. Res. 95 (5), 459–470. 10.1161/01.RES.0000141146.95728.da 15345668 PMC2810618

[B4] Artap S. Manderfield L. J. Smith C. L. Poleshko A. Aghajanian H. See K. (2018). Endocardial Hippo signaling regulates myocardial growth and cardiogenesis. Dev. Biol. 440 (1), 22–30. 10.1016/j.ydbio.2018.04.026 29727635 PMC5989000

[B5] Beermann J. Piccoli M. Viereck J. Thum T. (2016). Non-coding RNAs in development and disease: Background, mechanisms, and therapeutic Approaches. Physiol. Rev. 96 (4), 1297–1325. 10.1152/physrev.00041.2015 27535639

[B6] Bornhorst D. Xia P. Nakajima H. Dingare C. Herzog W. Lecaudey V. (2019). Biomechanical signaling within the developing zebrafish heart attunes endocardial growth to myocardial chamber dimensions. Nat. Commun. 10 (1), 4113. 10.1038/s41467-019-12068-x 31511517 PMC6739419

[B7] Braga L. Ali H. Secco I. Giacca M. (2021). Non-coding RNA therapeutics for cardiac regeneration. Cardiovasc. Res. 117 (3), 674–693. 10.1093/cvr/cvaa071 32215566 PMC7898953

[B8] Brozzi F. Regazzi R. (2021). Circular RNAs as novel regulators of β-cell functions under physiological and pathological Conditions. Int. J. Mol. Sci. 22 (4), 1503. 10.3390/ijms22041503 33546109 PMC7913224

[B9] Cai B. Ma W. Wang X. Sukhareva N. Hua B. Zhang L. (2020). Targeting LncDACH1 promotes cardiac repair and regeneration after myocardium infarction. Cell Death Differ. 27 (7), 2158–2175. 10.1038/s41418-020-0492-5 31969690 PMC7308407

[B10] Chaulk S. G. Lattanzi V. J. Hiemer S. E. Fahlman R. P. Varelas X. (2014). The Hippo pathway effectors TAZ/YAP regulate dicer expression and microRNA biogenesis through Let-7. J. Biol. Chem. 289 (4), 1886–1891. 10.1074/jbc.C113.529362 24324261 PMC3900939

[B11] Chen B. Dragomir M. P. Yang C. Li Q. Horst D. Calin G. A. (2022). Targeting non-coding RNAs to overcome cancer therapy resistance. Signal Transduct. Target. Ther. 7 (1), 121. 10.1038/s41392-022-00975-3 35418578 PMC9008121

[B12] Chen Z. Friedrich G. A. Soriano P. (1994). Transcriptional enhancer factor 1 disruption by a retroviral gene trap leads to heart defects and embryonic lethality in mice. Genes. Dev. 8 (19), 2293–2301. 10.1101/gad.8.19.2293 7958896

[B13] Christoffels V. Jensen B. (2020). Cardiac Morphogenesis: Specification of the four-Chambered heart. Cold Spring Harb. Perspect. Biol. 12 (10), a037143. 10.1101/cshperspect.a037143 31932321 PMC7528854

[B14] Dey A. Varelas X. Guan K. (2020). Targeting the Hippo pathway in cancer, fibrosis, wound healing and regenerative medicine. Nat. Rev. Drug Discov. 19 (7), 480–494. 10.1038/s41573-020-0070-z 32555376 PMC7880238

[B15] Diez-Cuñado M. Wei K. Bushway P. J. Maurya M. R. Perera R. Subramaniam S. (2018). miRNAs that induce human cardiomyocyte proliferation Converge on the hippo pathway. Cell Rep. 23 (7), 2168–2174. 10.1016/j.celrep.2018.04.049 29768213 PMC6261450

[B16] Ding H. Jiang M. Lau C. W. Luo J. Chan A. M. Wang L. (2023). Curaxin CBL0137 inhibits endothelial inflammation and atherogenesis via suppression of the Src-YAP signalling axis. Br. J. Pharmacol. 180 (8), 1168–1185. 10.1111/bph.16007 36495259

[B17] Donde M. J. Rochussen A. M. Kapoor S. Taylor A. I. (2022). Targeting non-coding RNA family members with artificial endonuclease XNAzymes. Commun. Biol. 5 (1), 1010. 10.1038/s42003-022-03987-5 36153384 PMC9509326

[B18] Encode (2012). An integrated encyclopedia of DNA elements in the human genome. Nature 489 (7414), 57–74. 10.1038/nature11247 22955616 PMC3439153

[B19] Francou A. De Bono C. Kelly R. G. (2017). Epithelial tension in the second heart field promotes mouse heart tube elongation. Nat. Commun. 8, 14770. 10.1038/ncomms14770 28357999 PMC5379109

[B20] Fukui H. Miyazaki T. Chow R. W. Ishikawa H. Nakajima H. Vermot J. (2018). Hippo signaling determines the number of venous pole cells that originate from the anterior lateral plate mesoderm in zebrafish. Elife 7, e29106. 10.7554/eLife.29106 29809141 PMC5995544

[B21] Fukui H. Terai K. Nakajima H. Chiba A. Fukuhara S. Mochizuki N. (2014). S1P-Yap1 signaling regulates endoderm formation required for cardiac precursor cell migration in zebrafish. Dev. Cell. 31 (1), 128–136. 10.1016/j.devcel.2014.08.014 25313964

[B22] Gan M. Zhang S. Fan Y. Tan Y. Guo Z. Chen L. (2020a). The expression of microRNA in adult rat heart with Isoproterenol-induced cardiac hypertrophy. Cells 9 (5), 1173. 10.3390/cells9051173 32397324 PMC7290591

[B23] Gan W. Dai X. Dai X. Xie J. Yin S. Zhu J. (2020b). LATS suppresses mTORC1 activity to directly coordinate Hippo and mTORC1 pathways in growth control. Nat. Cell Biol. 22 (2), 246–256. 10.1038/s41556-020-0463-6 32015438 PMC7076906

[B24] Ghafouri-Fard S. Abak A. Talebi S. F. Shoorei H. Branicki W. Taheri M. (2021). Role of miRNA and lncRNAs in organ fibrosis and aging. Biomed. Pharmacother. = Biomedecine Pharmacother. 143, 112132. 10.1016/j.biopha.2021.112132 34481379

[B25] Ghafouri-Fard S. Poornajaf Y. Hussen B. M. Tavakkoli Avval S. Taheri M. Mokhtari M. (2023). Deciphering the role of Hippo pathway in lung cancer. Pathology, Res. Pract. 243, 154339. 10.1016/j.prp.2023.154339 36736143

[B26] Gupta M. Kogut P. Davis F. J. Belaguli N. S. Schwartz R. J. Gupta M. P. (2001). Physical interaction between the MADS box of serum response factor and the TEA/ATTS DNA-binding domain of transcription enhancer factor-1. J. Biol. Chem. 276 (13), 10413–10422. 10.1074/jbc.M008625200 11136726

[B27] Hara H. Takeda N. Kondo M. Kubota M. Saito T. Maruyama J. (2018). Discovery of a small molecule to increase cardiomyocytes and protect the heart after ischemic injury. JACC. Basic Transl. Sci. 3 (5), 639–653. 10.1016/j.jacbts.2018.07.005 30456335 PMC6234526

[B28] Hazra R. Brine L. Garcia L. Benz B. Chirathivat N. Shen M. M. (2022). Platr4 is an early embryonic lncRNA that exerts its function downstream on cardiogenic mesodermal lineage commitment. Dev. Cell. 57 (21), 2450–2468.e7. 10.1016/j.devcel.2022.10.002 36347239 PMC9680017

[B29] He L. Zhang Q. Jiang D. Zhang Y. Wei Y. Yang Y. (2022). Zebrafish Foxc1a controls ventricular chamber maturation by directly regulating wwtr1 and nkx2.5 expression. J. Genet. genomics = Yi chuan xue bao. 49 (6), 559–568. 10.1016/j.jgg.2021.12.002 34923164

[B30] Hsu H. Estarás C. Huang L. Jones K. A. (2018). Specifying the anterior primitive streak by Modulating YAP1 levels in human pluripotent stem cells. Stem Cell Rep. 11 (6), 1357–1364. 10.1016/j.stemcr.2018.10.013 PMC6294113 30449705

[B31] Hu J. Liu T. Zhang Z. Xu Y. Zhu F. (2019). Oxidized low-density lipoprotein promotes vascular endothelial cell dysfunction by stimulating miR-496 expression and inhibiting the Hippo pathway effector YAP. Cell Biol. Int. 43 (5), 528–538. 10.1002/cbin.11120 30811087 PMC6850352

[B32] Jin X. Yu W. Ye P. (2022). MiR-125b enhances doxorubicin-induced cardiotoxicity by suppressing the nucleus-cytoplasmic translocation of YAP via targeting STARD13. Environ. Toxicol. 37 (4), 730–740. 10.1002/tox.23438 34921586

[B33] Johnson R. Halder G. (2014). The two faces of Hippo: targeting the Hippo pathway for regenerative medicine and cancer treatment. Nat. Rev. Drug Discov. 13 (1), 63–79. 10.1038/nrd4161 24336504 PMC4167640

[B34] Justice R. W. Zilian O. Woods D. F. Noll M. Bryant P. J. (1995). The Drosophila tumor suppressor gene warts encodes a homolog of human myotonic dystrophy kinase and is required for the control of cell shape and proliferation. Dev 9 (5), 534–546. 10.1101/gad.9.5.534 7698644

[B35] Kastan N. Gnedeva K. Alisch T. Petelski A. A. Huggins D. J. Chiaravalli J. (2021). Small-molecule inhibition of Lats kinases may promote Yap-dependent proliferation in postmitotic mammalian tissues. Nat. Commun. 12 (1), 3100. 10.1038/s41467-021-23395-3 34035288 PMC8149661

[B36] Lai J. Collins M. M. Uribe V. Jiménez-Amilburu V. Günther S. Maischein H. M. (2018). The Hippo pathway effector Wwtr1 regulates cardiac wall maturation in zebrafish. Development 145 (10), dev159210. 10.1242/dev.159210 29773645

[B37] Li C. Ni Y. Xu H. Xiang Q. Zhao Y. Zhan J. (2021). Roles and mechanisms of exosomal non-coding RNAs in human health and diseases. Signal Transduct. Target. Ther. 6 (1), 383. 10.1038/s41392-021-00779-x 34753929 PMC8578673

[B38] Li H. Trager L. E. Liu X. Hastings M. H. Xiao C. Guerra J. (2022a). lncExACT1 and DCHS2 regulate physiological and pathological cardiac growth. Circulation 145 (16), 1218–1233. 10.1161/CIRCULATIONAHA.121.056850 35114812 PMC9056949

[B39] Li H. Zhu J. Xu Y. Mou F. Shan X. Wang Q. (2022b). Notoginsenoside R1-loaded mesoporous silica nanoparticles targeting the site of injury through inflammatory cells improves heart repair after myocardial infarction. Redox Biol. 54, 102384. 10.1016/j.redox.2022.102384 35777198 PMC9287735

[B40] Li P. Chen Y. Mak K. K. Wong C. K. Wang C. C. Yuan P. (2013). Functional role of Mst1/Mst2 in embryonic stem cell differentiation. PLoS One 8 (11), e79867. 10.1371/journal.pone.0079867 24224013 PMC3818222

[B41] Li X. Feng S. Luo Y. Long K. Lin Z. Ma J. (2018). Expression profiles of microRNAs in oxidized low-density lipoprotein-stimulated RAW 264.7 cells. Animal 54 (2), 99–110. 10.1007/s11626-017-0225-3 29322359

[B42] Lin Z. Guo H. Cao Y. Zohrabian S. Zhou P. Ma Q. (2016). Acetylation of VGLL4 regulates hippo-YAP signaling and postnatal cardiac growth. Dev. Cell. 39 (4), 466–479. 10.1016/j.devcel.2016.09.005 27720608 PMC5121000

[B43] Lin Z. Zhou P. von Gise A. Gu F. Ma Q. Chen J. (2015). Pi3kcb links Hippo-YAP and PI3K-AKT signaling pathways to promote cardiomyocyte proliferation and survival. Circ. Res. 116 (1), 35–45. 10.1161/CIRCRESAHA.115.304457 25249570 PMC4282610

[B44] Liu J. Xu L. Zhan X. (2020). LncRNA MALAT1 regulates diabetic cardiac fibroblasts through the Hippo-YAP signaling pathway. Biochem. cell Biol. = Biochimie Biol. Cell. 98 (5), 537–547. 10.1139/bcb-2019-0434 32069074

[B45] Liu R. Lee J. Kim B. S. Wang Q. Buxton S. K. Balasubramanyam N. (2017). Tead1 is required for maintaining adult cardiomyocyte function, and its loss results in lethal dilated cardiomyopathy. JCI insight 2 (17), e93343. 10.1172/jci.insight.93343 28878117 PMC5621883

[B46] Liu S. Li K. Wagner Florencio L. Tang L. Heallen T. R. Leach J. P. (2021). Gene therapy knockdown of Hippo signaling induces cardiomyocyte renewal in pigs after myocardial infarction. Sci. Transl. Med. 13 (600), eabd6892. 10.1126/scitranslmed.abd6892 34193613 PMC9476348

[B47] Loganathan T. Doss C. G. P. (2023). Non-coding RNAs in human health and disease: potential function as biomarkers and therapeutic targets. Funct. Integr. Genomics 23 (1), 33. 10.1007/s10142-022-00947-4 36625940 PMC9838419

[B48] Ma C. Peng P. Zhou Y. Liu T. Wang L. Lu C. (2020). MicroRNA‑93 promotes angiogenesis and attenuates remodeling via inactivation of the Hippo/Yap pathway by targeting Lats2 after myocardial infarctionω. Mol. Med. Rep. 22 (1), 483–493. 10.3892/mmr.2020.11085 32319642 PMC7248469

[B49] Ma S. Dong Z. (2019). Melatonin attenuates cardiac reperfusion stress by improving OPA1-related mitochondrial fusion in a Yap-hippo pathway-dependent manner. J. Cardiovasc. Pharmacol. 73 (1), 27–39. 10.1097/FJC.0000000000000626 30418242 PMC6319588

[B50] Maeda T. Gupta M. P. Stewart A. F. R. (2002). TEF-1 and MEF2 transcription factors interact to regulate muscle-specific promoters. Biochem. Biophys. Res. Commun. 294 (4), 791–797. 10.1016/S0006-291X(02)00556-9 12061776

[B51] Manning S. A. Kroeger B. Harvey K. F. (2020). The regulation of Yorkie, YAP and TAZ: new insights into the Hippo pathway. Dev. Camb. Engl. 147 (8), dev179069. 10.1242/dev.179069 32341025

[B52] Mao Y. Jiang L. (2021). MiR-200c-3p promotes ox-LDL-induced endothelial to mesenchymal transition in human umbilical vein endothelial cells through SMAD7/YAP pathway. J. physiological Sci. JPS 71 (1), 30. 10.1186/s12576-021-00815-z PMC10717414 34525946

[B53] Matsui Y. Nakano N. Shao D. Gao S. Luo W. Hong C. (2008). Lats2 is a negative regulator of myocyte size in the heart. Circ. Res. 103 (11), 1309–1318. 10.1161/CIRCRESAHA.108.180042 18927464 PMC2775813

[B54] Misra J. R. Irvine K. D. (2018). The hippo signaling network and its biological functions. Annu. Rev. Genet. 52, 65–87. 10.1146/annurev-genet-120417-031621 30183404 PMC6322405

[B55] Morikawa Y. Heallen T. Leach J. Xiao Y. Martin J. F. (2017). Dystrophin-glycoprotein complex sequesters Yap to inhibit cardiomyocyte proliferation. Nature 547 (7662), 227–231. 10.1038/nature22979 28581498 PMC5528853

[B56] Morikawa Y. Zhang M. Heallen T. Leach J. Tao G. Xiao Y. (2015). Actin cytoskeletal remodeling with protrusion formation is essential for heart regeneration in Hippo-deficient mice. Sci. Signal. 8 (375), ra41. 10.1126/scisignal.2005781 25943351 PMC4442128

[B57] Moya I. M. Halder G. (2019). Hippo-YAP/TAZ signalling in organ regeneration and regenerative medicine. Nat. Rev. Mol. cell Biol. 20 (4), 211–226. 10.1038/s41580-018-0086-y 30546055

[B58] Murphy S. A. Miyamoto M. Kervadec A. Kannan S. Tampakakis E. Kambhampati S. (2021). PGC1/PPAR drive cardiomyocyte maturation at single cell level via YAP1 and SF3B2. Nat. Commun. 12 (1), 1648. 10.1038/s41467-021-21957-z 33712605 PMC7955035

[B59] Ni J. Liu Y. Wang K. Wu M. Kang L. Sha D. (2020). Trophoblast stem-cell-derived exosomes improve doxorubicin-induced dilated cardiomyopathy by Modulating the let-7i/YAP pathway. Nucleic acids. 22, 948–956. 10.1016/j.omtn.2020.10.014 33294288 PMC7680701

[B60] Nojima T. Proudfoot N. J. (2022). Mechanisms of lncRNA biogenesis as revealed by nascent transcriptomics. Nat. Rev. Mol. cell Biol. 23 (6), 389–406. 10.1038/s41580-021-00447-6 35079163

[B61] Nugroho A. B. Stafford N. Zi M. Prehar S. Potter R. Kwon D. (2022). Micro RNA-411 expression improves cardiac phenotype Following myocardial infarction in mice. JACC-Basic Transl. Sci. 7 (9), 859–875. 10.1016/j.jacbts.2022.05.008 36317138 PMC9617134

[B62] O'Brien J. Hayder H. Zayed Y. Peng C. (2018). Overview of MicroRNA biogenesis, mechanisms of actions, and circulation. Front. Endocrinol. 9, 402. 10.3389/fendo.2018.00402 PMC6085463 30123182

[B63] O'Donnell A. Yutzey K. E. (2020). Mechanisms of heart valve development and disease. Dev. Camb. Engl. 147 (13), dev183020. 10.1242/dev.183020 PMC7338271 32620577

[B64] Panciera T. Azzolin L. Cordenonsi M. Piccolo S. (2017). Mechanobiology of YAP and TAZ in physiology and disease. Nat. Rev. Mol. cell Biol. 18 (12), 758–770. 10.1038/nrm.2017.87 28951564 PMC6192510

[B65] Park S. Choe M. Yeo H. Han H. Kim J. Chang W. (2018). Yes-associated protein mediates human embryonic stem cell-derived cardiomyocyte proliferation: involvement of epidermal growth factor receptor signaling. J. Cell. Physiol. 233 (10), 7016–7025. 10.1002/jcp.26625 29693249

[B66] Piccolo S. Dupont S. Cordenonsi M. (2014). The biology of YAP/TAZ: hippo signaling and beyond. Physiol. Rev. 94 (4), 1287–1312. 10.1152/physrev.00005.2014 25287865

[B67] Piquer-Gil M. Domenech-Dauder S. Sepúlveda-Gómez M. Machí-Camacho C. Braza-Boïls A. Zorio E. (2022). Non coding RNAs as regulators of Wnt/β-catenin and hippo pathways in arrhythmogenic cardiomyopathy. Biomedicines 10 (10), 2619. 10.3390/biomedicines10102619 36289882 PMC9599412

[B68] Ragni C. V. Diguet N. Le Garrec J. F. Novotova M. Resende T. P. Pop S. (2017). Amotl1 mediates sequestration of the Hippo effector Yap1 downstream of Fat4 to restrict heart growth. Nat. Commun. 8, 14582. 10.1038/ncomms14582 28239148 PMC5333361

[B69] Rai A. K. Lee B. Hebbard C. Uchida S. Garikipati V. N. S. (2021). Decoding the complexity of circular RNAs in cardiovascular disease. Pharmacol. Res. 171, 105766. 10.1016/j.phrs.2021.105766 34271160

[B70] Sánchez-Sánchez R. Reinal I. Peiró-Molina E. Buigues M. Tejedor S. Hernándiz A. (2022). MicroRNA-4732-3p is Dysregulated in Breast cancer patients with cardiotoxicity, and its therapeutic delivery protects the heart from doxorubicin-induced oxidative stress in rats. Antioxidants Basel, Switz. 11 (10), 1955. 10.3390/antiox11101955 PMC9599023 36290678

[B71] Shah A. M. Giacca M. (2022). Small non-coding RNA therapeutics for cardiovascular disease. Eur. Heart J. 43 (43), 4548–4561. 10.1093/eurheartj/ehac463 36106499 PMC9659475

[B72] Shao Y. Li M. Yu Q. Gong M. Wang Y. Yang X. (2022). CircRNA CDR1as promotes cardiomyocyte apoptosis through activating hippo signaling pathway in diabetic cardiomyopathy. Eur. J. Pharmacol. 922, 174915. 10.1016/j.ejphar.2022.174915 35339477

[B73] She G. Du J. Wu W. Pu T. Zhang Y. Bai R. (2023). Hippo pathway activation mediates chemotherapy-induced anti-cancer effect and cardiomyopathy through causing mitochondrial damage and dysfunction. Theranostics 13 (2), 560–577. 10.7150/thno.79227 36632235 PMC9830444

[B74] Shen S. Guo X. Yan H. Lu Y. Ji X. Li L. (2015). A miR-130a-YAP positive feedback loop promotes organ size and tumorigenesis. Cell Res. 25 (9), 997–1012. 10.1038/cr.2015.98 26272168 PMC4559818

[B75] Singh A. Ramesh S. Cibi D. M. Yun L. S. Li J. Li L. (2016). Hippo signaling mediators Yap and Taz are required in the epicardium for coronary Vasculature development. Cell Rep. 15 (7), 1384–1393. 10.1016/j.celrep.2016.04.027 27160901 PMC4871746

[B76] Statello L. Guo C. Chen L. Huarte M. (2021). Gene regulation by long non-coding RNAs and its biological functions. Nat. Rev. Mol. cell Biol. 22 (2), 96–118. 10.1038/s41580-020-00315-9 33353982 PMC7754182

[B77] Struhl K. (2007). Transcriptional noise and the fidelity of initiation by RNA polymerase II. Nat. Struct. Mol. Biol. 14 (2), 103–105. 10.1038/nsmb0207-103 17277804

[B78] Sygitowicz G. Sitkiewicz D. (2022). Involvement of circRNAs in the development of heart failure. Int. J. Mol. Sci. 23 (22), 14129. 10.3390/ijms232214129 36430607 PMC9697219

[B79] Tian Y. Liu Y. Wang T. Zhou N. Kong J. Chen L. (2015). A microRNA-Hippo pathway that promotes cardiomyocyte proliferation and cardiac regeneration in mice. Sci. Transl. Med. 7 (279), 279ra38. 10.1126/scitranslmed.3010841 PMC6295313 25787764

[B80] Torrini C. Cubero R. J. Dirkx E. Braga L. Ali H. Prosdocimo G. (2019). Common regulatory pathways mediate activity of MicroRNAs inducing cardiomyocyte proliferation. Cell Rep. 27 (9), 2759–2771. 10.1016/j.celrep.2019.05.005 31141697 PMC6547019

[B81] Triastuti E. Nugroho A. B. Zi M. Prehar S. Kohar Y. S. Bui T. A. (2019). Pharmacological inhibition of Hippo pathway, with the novel kinase inhibitor XMU-MP-1, protects the heart against adverse effects during pressure overload. Br. J. Pharmacol. 176 (20), 3956–3971. 10.1111/bph.14795 31328787 PMC6811740

[B82] Tsao C. W. Aday A. W. Almarzooq Z. I. Anderson C. A. M. Arora P. Avery C. L. (2023). Heart disease and Stroke Statistics-2023 Update: a Report from the American heart association. Circulation 147 (8), e93–e621. 10.1161/CIR.0000000000001123 36695182 PMC12135016

[B83] Tumaneng K. Schlegelmilch K. Russell R. C. Yimlamai D. Basnet H. Mahadevan N. (2012). YAP mediates crosstalk between the Hippo and PI(3)K–TOR pathways by suppressing PTEN via miR-29. Nat. Cell Biol. 14 (12), 1322–1329. 10.1038/ncb2615 23143395 PMC4019071

[B84] Varelas X. (2014). The Hippo pathway effectors TAZ and YAP in development, homeostasis and disease. Dev. Camb. Engl. 141 (8), 1614–1626. 10.1242/dev.102376 24715453

[B85] Vite A. Zhang C. Yi R. Emms S. Radice G. L. (2018). α-Catenin-dependent cytoskeletal tension controls Yap activity in the heart. Dev. Camb. Engl. 145 (5), dev149823. 10.1242/dev.149823 PMC5868989 29467248

[B86] von Gise A. Lin Z. Schlegelmilch K. Honor L. B. Pan G. M. Buck J. N. (2012). YAP1, the nuclear target of Hippo signaling, stimulates heart growth through cardiomyocyte proliferation but not hypertrophy. Proc. Natl. Acad. Sci. U. S. A. 109 (7), 2394–2399. 10.1073/pnas.1116136109 22308401 PMC3289361

[B87] Wang J. Liu S. Heallen T. Martin J. F. (2018). The Hippo pathway in the heart: pivotal roles in development, disease, and regeneration. Nat. Rev. Cardiol. 15 (11), 672–684. 10.1038/s41569-018-0063-3 30111784

[B88] Wang K. Yeh Y. Nguyen P. Limqueco E. Lopez J. Thorossian S. (2016). Flow-dependent YAP/TAZ activities regulate endothelial phenotypes and atherosclerosis. Proc. Natl. Acad. Sci. U. S. A. 113 (41), 11525–11530. 10.1073/pnas.1613121113 27671657 PMC5068257

[B89] Wang L. Choi K. Su T. Li B. Wu X. Zhang R. (2022). Multiphase coalescence mediates Hippo pathway activation. Cell 185 (23), 4376–4393.e18. 10.1016/j.cell.2022.09.036 36318920 PMC9669202

[B90] Wilson K. D. Ameen M. Guo H. Abilez O. J. Tian L. Mumbach M. R. (2020). Endogenous Retrovirus-derived lncRNA BANCR promotes cardiomyocyte migration in humans and non-human primates. Dev. Cell. 54 (6), 694–709. 10.1016/j.devcel.2020.07.006 32763147 PMC7529962

[B91] Wu N. Xu J. Du W. W. Li X. Awan F. M. Li F. (2021). YAP circular RNA, circYap, attenuates cardiac fibrosis via binding with tropomyosin-4 and gamma-actin decreasing actin polymerization. Mol. Ther. J. Am. Soc. Gene Ther. 29 (3), 1138–1150. 10.1016/j.ymthe.2020.12.004 PMC7934790 33279723

[B92] Wu Y. Guo X. Han T. Feng K. Zhang P. Xu Y. (2022). Cmarr/miR-540-3p axis promotes cardiomyocyte maturation transition by orchestrating Dtna expression. Nucleic acids. 29, 481–497. 10.1016/j.omtn.2022.07.022 36035750 PMC9382425

[B93] Xiao Y. Hill M. C. Zhang M. Martin T. J. Morikawa Y. Wang S. (2018). Hippo signaling plays an essential role in cell state transitions during cardiac fibroblast development. Dev. Cell. 45 (2), 153–169. 10.1016/j.devcel.2018.03.019 29689192 PMC5947860

[B94] Xie Y. Wang Q. Gao N. Wu F. Lan F. Zhang F. (2020). MircroRNA-10b promotes human embryonic stem cell-derived cardiomyocyte proliferation via novel target gene LATS1. Nucleic acids. 19, 437–445. 10.1016/j.omtn.2019.11.026 31902743 PMC6948266

[B95] Xin M. Kim Y. Sutherland L. B. Murakami M. Qi X. Mcanally J. (2013). Hippo pathway effector Yap promotes cardiac regeneration. Proc. Natl. Acad. Sci. U. S. A. 110 (34), 13839–13844. 10.1073/pnas.1313192110 23918388 PMC3752208

[B96] Xin M. Kim Y. Sutherland L. B. Qi X. Mcanally J. Schwartz R. J. (2011). Regulation of insulin-like growth factor signaling by Yap governs cardiomyocyte proliferation and embryonic heart size. Sci. Signal. 4 (196), ra70. 10.1126/scisignal.2002278 22028467 PMC3440872

[B97] Xu F. Yang J. Shang J. Lan F. Li M. Shi L. (2019). MicroRNA-302d promotes the proliferation of human pluripotent stem cell-derived cardiomyocytes by inhibiting LATS2 in the Hippo pathway. Clin. Sci. (Lond). 133 (13), 1387–1399. 10.1042/CS20190099 31239293

[B98] Xu X. Zhang Q. Song H. Ao Z. Li X. Cheng C. (2018). Effects of artemisinin on ventricular arrhythmias in response to left ventricular afterload increase and microRNA expression profiles in Wistar rats. PeerJ 6, e6110. 10.7717/peerj.6110 30595983 PMC6304267

[B99] Yan S. Peng Y. Lu J. Shakil S. Shi Y. Crossman D. K. (2022). Differential requirement for DICER1 activity during the development of mitral and tricuspid valves. J. Cell Sci. 135 (17), jcs259783. 10.1242/jcs.259783 35946425 PMC9482344

[B100] Yang Y. Del Re D. P. Nakano N. Sciarretta S. Zhai P. Park J. (2015). miR-206 mediates YAP-induced cardiac hypertrophy and survival. Circ. Res. 117 (10), 891–904. 10.1161/CIRCRESAHA.115.306624 26333362 PMC4747867

[B101] Yu F. Zhao B. Guan K. (2015). Hippo pathway in organ size control, tissue homeostasis, and cancer. Cell. 163 (4), 811–828. 10.1016/j.cell.2015.10.044 26544935 PMC4638384

[B102] Zhang C. Niu K. Lian P. Hu Y. Shuai Z. Gao S. (2021). Pathological Bases and clinical application of long noncoding RNAs in cardiovascular diseases. Hypertens. 78 (1), 16–29. 10.1161/HYPERTENSIONAHA.120.16752 34058852

[B103] Zhang H. Liu S. Dong T. Yang J. Xie Y. Wu Y. (2016). Profiling of differentially expressed microRNAs in arrhythmogenic right ventricular cardiomyopathy. Sci. Rep. 6, 28101. 10.1038/srep28101 27307080 PMC4910108

[B104] Zheng Y. Pan D. (2019). The hippo signaling pathway in development and disease. Dev. Cell. 50 (3), 264–282. 10.1016/j.devcel.2019.06.003 31386861 PMC6748048

[B105] Zhou Q. Li L. Zhao B. Guan K. (2015). The hippo pathway in heart development, regeneration, and diseases. Circ. Res. 116 (8), 1431–1447. 10.1161/CIRCRESAHA.116.303311 25858067 PMC4394208

